# CircRNA_100367 regulated the radiation sensitivity of esophageal squamous cell carcinomas through miR-217/Wnt3 pathway

**DOI:** 10.18632/aging.102580

**Published:** 2019-12-18

**Authors:** Junqi Liu, Nannan Xue, Yuexin Guo, Kerun Niu, Liang Gao, Song Zhang, Hao Gu, Xin Wang, Di Zhao, Ruitai Fan

**Affiliations:** 1 Department of Radiation Oncology, The First Affiliated Hospital of Zhengzhou University, Erqi, Zhengzhou 450000, China; 2 Department of Molecular and Radiation Oncology, German Cancer Research Center (DKFZ), Heidelberg 69120, Germany; 3 Center of Experimental Orthopaedics, Saarland University Medical Center, Kirrberger Strasse, Homburg 66421, Germany; 4 Endocrinology Department, The First Affiliated Hospital of Zhengzhou University, Zhengzhou 450000, China

**Keywords:** circRNA_100367, miR-217, Wnt3, radiation sensitivity, esophageal squamous cell carcinoma

## Abstract

Background: Circular RNAs (circRNAs) play important roles in regulating the radioresistance of esophageal squamous cell carcinoma (ESCC). This study aimed to determine the role of hsa_circRNA_100367 in regulating radioresistance of ESCC.

Results: Higher expression and potency of endothelial to mesenchymal transformation (EMT) was found in radioresistant ESCC cells (KYSE-150R) than in ESCC cells (KYSE-150). Silencing circRNA_100367 inhibited the proliferation and migration of KYSE-150R cells, and decreased the expression of β-catenin (an important molecule in Wnt pathway) in KYSE-150R cells. Additionally, circRNA_100367 bound to miR-217, and miR-217 targeted Wnt3. Low Wnt3 expression was associated with the short survival time in patients with ESCC and Wnt3 knockdown inhibited the proliferation and migration of KYSE-150R cells. CircRNA_100367 enhanced the radioresistance of KYSE-150R cells through miR-217/Wnt3 pathway. *In vivo*, circRNA_100367 silence reduced the growth of KYSE-150R cells under radiation.

Conclusion: Our results revealed that circRNA_100367 attenuated radioresistance of ESCC through miR-217/Wnt3 pathway.

Methods: CircRNAs related with the radioresistance of ESCC were analyzed by hierarchical cluster analysis. The relationship between circRNA_100367 and miR-217, Wnt3 was detected by RNA immunoprecipitation (RIP), RNA pull-down and luciferase reporte assays. The proliferation and migration ESCC cells were detected by MTT, Transwell and colony formation assays.

## INTRODUCTION

Esophageal squamous cell carcinoma (ESCC), as the predominant form of esophageal cancer and accounting for about 88% of esophageal cancer, is the sixth leading cause of cancer death in the world and the second most deadly cancer in China [1, 2]. Preoperative radiotherapy is a vital treatment of ESCC in clinic [3, 4], but it is not effective for all patients with ESCC because of the occurrence of radioresistance of ESCC cells [5]. Studies have shown that the occurrence of radioresistance of ESCC cells is related with the abnormal expression of genes [6, 7]. Therefore, it is important to find abnormally expressed genes that related with the radiation sensitivity of ESCC, which will provide potential therapeutic targets for the treatment of ESCC.

Circular RNAs (circRNAs) are universal diverse endogenous noncoding RNAs that have covalently linked ends which can be acted as microRNA (miRNA) sponges to regulate gene expressions in many cancers [8]. Studies have reported that circRNAs are abnormally expressed in various cancer cells, such as cervical cancer cell, hepatic stellate cells and ESCC cells, which play important roles in regulating the radiation sensitivity of these cancers [9–11]. However, there are few studies focused on a specific circRNA in regulating the radiation sensitivity of ESCC. Recently, the expressions of 3752 circRNAs are detected in ESCC cell line KYSE-150 and radioresistant ESCC cell KYSE-150R, and hsa_circRNA_100367 (gene Symbol DDB1 and CUL4 associated factor 8 (DCAF8), also named hsa_circRNA_0014879) is found to be highly expressed in KYSE-150R cell line [12]. Therefore, circRNA_100367 may play a vital role in regulating the radiation sensitivity of ESCC. However, the underlying mechanism of circRNA_100367 in the radiation sensitivity of ESCC is still not clear.

Studies have reported that circRNA exert its function in ESCC through competitive binding with miRNAs. For example, circRNA VRK1 inhibited the progression of ESCC through regulating miR-624-3p/ phosphatase and tensin homolog (PTEN) signaling pathway [11]. So, finding the downstream miRNAs/proteins that regulated by circRNA_100367 is important to reveal the underlying mechanism of circRNA_100367 mediated radiation sensitivity of ESCC. miR-217 is a widely expressed miRNA and acts as a tumor suppressor gene DCAF or oncogene in various cancers [13–15]. In addition, it has been reported that miR-217 can inhibit the progression of ESCC [16], and enhance the radiation sensitivity to doxorubicin in acute myeloid leukemia [17]. Base on the analysis of bioinformatics software (circinteractome), we found there are binding sties between circRNA_100367 and miR-217. Therefore, circRNA_100367 might regulate the radiation sensitivity of ESCC through sponging miR-217.

Previous studies have shown that the 3’-untranslated region (UTR) of target mRNAs can be bound by specific miRNAs to play important roles in the regulation of radiation sensitivity of ESCC [18, 19]. Wnt3 is a member of Wnt family, and is the key molecule of Wnt-β-catenin signaling pathway. The expression of Wnt3 can activate Wnt-β-catenin signaling pathway in liver cancer, gastric cancer, lung cancer, and ESCC [20–22], etc. Studies have shown that Wnt3 is up-regulated in cancer tissues, such as gastric cancer and lung cancer, and knockdown of Wnt3 can suppress the progression of cancers [20, 21]. Wnt3 is also reported to be associated with the tumorigenicity and poor prognosis of ESCC [22, 23]. Importantly, Wnt-β-catenin signaling is found to enhance radioresistance of ESCC [24]. Bioinformatics softwares predicted there are binding sites between miR-217 and Wnt3 3’UTR. However, whether circRNA_100367 can regulate the radiation sensitivity of ESCC through miR-217/Wnt3 pathway is still known. In the present study, we aimed to elucidate this question.

## RESULTS

### hsa_circRNA_100367 was highly expressed in human radioresistant esophageal cancer cell lines

**Figure 1:**
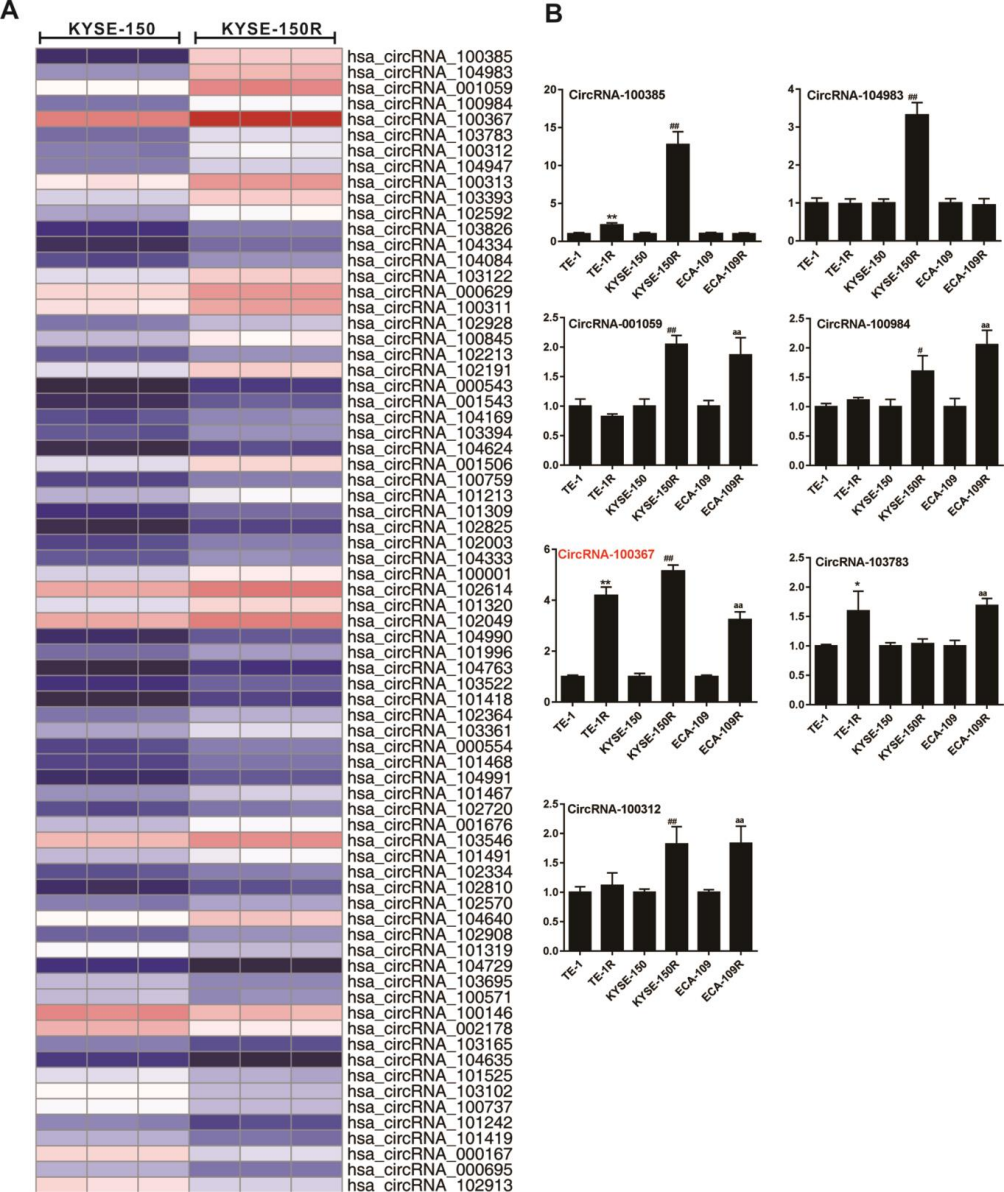
**Expression of hsa_circRNA_100367 in human radioresistant esophageal cancer cell lines.** (**A**) Hierarchical cluster analysis showed distinguishable circRNA expression profiles between KYSE-150 and KYSE-150R cells. (**B**) qRT-PCR detected circRNA_100385, circRNA_104983, circRNA_001059, circRNA_100984, circRNA_100367, circRNA_103783 and circRNA_100312 expressions in TE-1, TE-1R, KYSE-150, KYSE-150R, ECA-109, ECA-109R cells. *p<0.05, **p<0.01 vs. TE-1; #p<0.05, ^##^p<0.01 vs. KYSE-150; ^aa^p<0.01 vs ECA-109.

According to the online circRNA information in radioresistant esophageal cancer cells 12 [12], 74 differentially expressed circRNAs (FDR<0.01, fold change>2 or <0.5) were selected for circRNAs chips (Figure 1A). Then, hierarchical cluster analysis was performed to select the differentially expressed circRNAs (fold change>5 or < 0.2) between the KYSE-150 and KYSE-150R cells, and the expressions of those circRNAs were measured in different esophageal cancer cell lines (TE-1/TE-1R, KYSE-150/KYSE-150R, ECA-109/ECA-109R cells). As shown in Figure 1B, the expression trend of circRNA_100367 was consistent in TE-1/TE-1R, KYSE-150/KYSE-150R, and ECA-109/ECA-109R cells. Moreover, circRNA_100367 showed an increased expression trend with a most extent in KYSE-150/KYSE-150R cells. Therefore, circRNA_100367 was selected for further experiments.

### CircRNA_100367 exists in KYSE-150R cells with higher potency of endothelial to mesenchymal transformation (EMT)

**Figure 2:**
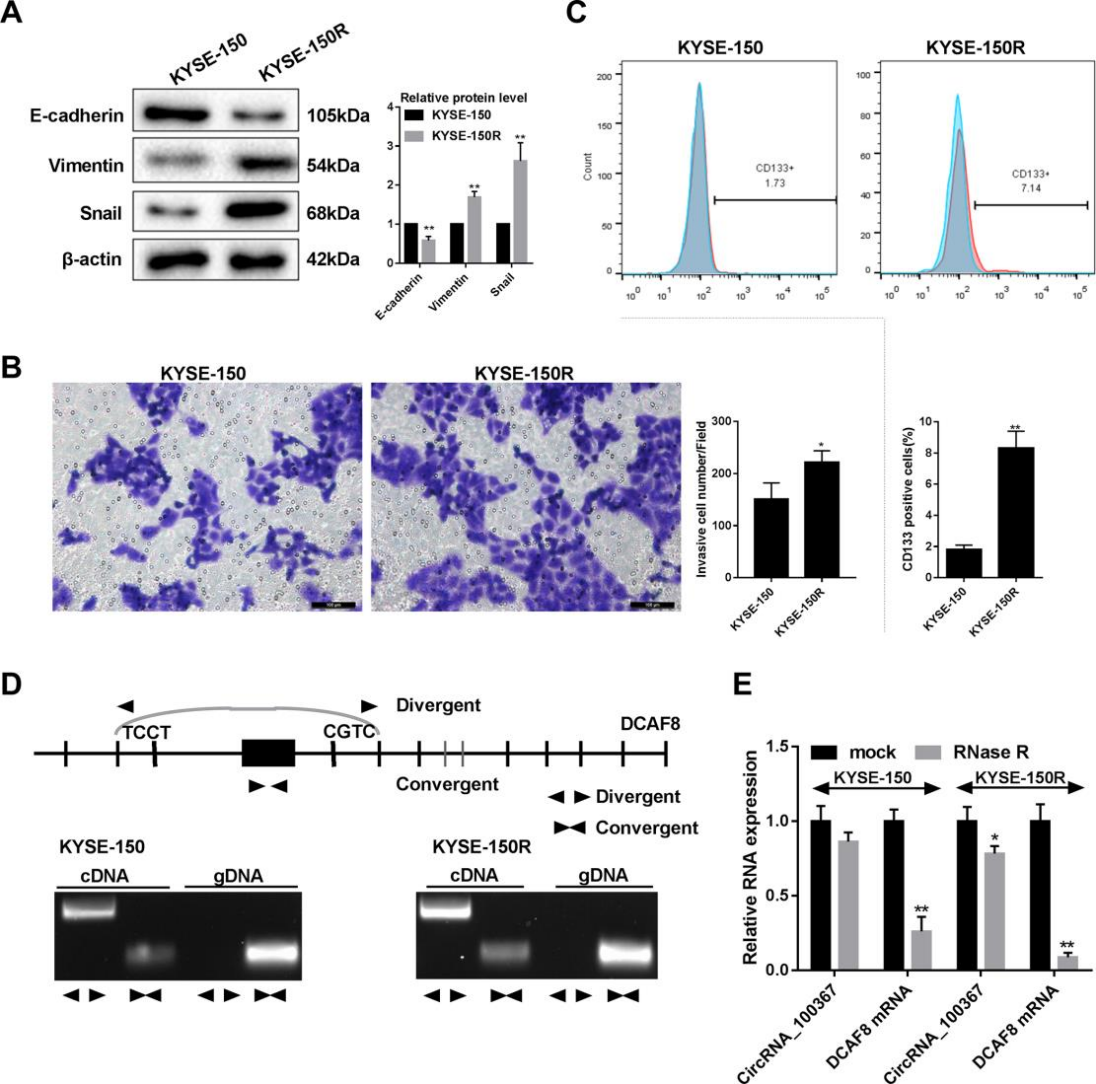
**CircRNA_100367 exists in KYSE-150R cells with higher potency of endothelial to mesenchymal transformation (EMT).** (**A**) The protein levels characteristic molecules (β-catenin, Vimentin, and Snail) of EMT in KYSE-150 and KYSE-150R cells were detected by western blot. (**B**) The migration of KYSE-150 and KYSE-150R cells were determined by transwell assay. (**C**) The CD133 positive cells of KYSE-150 and KYSE-150R cells were measured by flow cytometry. (**D**) Divergent primers and convergent primers were designed, and PCR product of cricRNA 100367 was detected in 1.5% agarose gel electrophoresis. (**E**) After the treatment of RNase R, circRNA_100367 and DCAF8 mRNA expressions in KYSE-150 and KYSE-150R cells were detected by qRT-PCR. *p<0.05, p<0.01 vs. KYSE-150 or mock.

As shown in Figure 2A, EMT-related molecule E-cadherin was decreased and vimentin and snail expressions were increased in KYSE-150R cells than that of KYSE-150 cells, indicating stronger molecular characteristics of EMT in KYSE-150R cells. Besides, the number of migrated KYSE-150R cells was greater than KYSE-150 cells (Figure 2B). The number of CD133+ cells in KYSE-150R cells was greater than that in KYSE-150 cells (Figure 2C). The role of circRNA_100367 was further determined in KYSE-150/KYSE-150R cells. The circular properties of cricRNA 100367 was identified with divergent primers and convergent primers (Figure 2D). Compared with mock KYSE-150R cells, circRNA_100367 expression was significantly down-regulated in RNase R-treated KYSE-150R cells. DCAF8 mRNA expression was significantly down-regulated in RNase R-treated KYSE-150 cells and KYSE-150R cells (Figure 2E). However, the degradation degree of circRNA_100367 was much lower than DCAF8 mRNA, indicating circRNA_100367 exists in esophageal squamous cell carcinomas.

### Silencing circRNA_100367 inhibited the proliferation and migration of KYSE-150R cells

**Figure 3:**
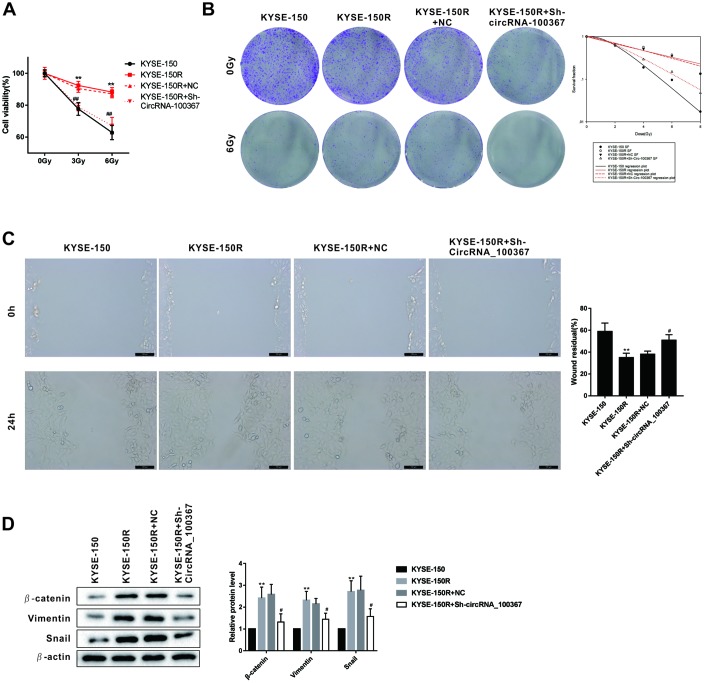
**Effects of circRNA_100367 on the proliferation and migration of KYSE-150R cells.** (**A**) KYSE-150R cells were transfected with Sh-circRNA_100367 (or NC) and then irradiated with 0, 3, and 6 Gy X-ray. Cell ability was detected by MTT assay. (**B**) KYSE-150R cells were transfected with Sh-circRNA_100367 (or NC) and then irradiated with different dose X-ray. The proliferation of cells was evaluated by colony formation assay and survival fraction was analyzed by multi-target single-hit model. (**C**) KYSE-150R cells were transfected with Sh-circRNA_100367 (or NC). The migration of cells was assessed by wound healing assay. (**D**) KYSE-150R cells were transfected with Sh-circRNA_100367 (or NC). The protein levels of β-catenin, Vimentin, and Snail were detected by western blot. NC: the negative control of Sh-circRNA_100367. **p<0.01 vs. KYSE-150; #p<0.05, ##p<0.01 vs. KYSE-150+NC.

Since circRNA_100367 was highly expressed in KYSE-150R cells, sh- circRNA_100367 was transfected into KYSE-150R cells to block the circRNA_100367 expression. As shown in Figure 3A, the viability of KYSE-150 and KYSE-150R cells was decreased with the increase of radiation dose. The viability of KYSE-150R cells was significantly decreased after the treatment of sh- circRNA_100367, indicating silencing circRNA_100367 enhanced the radiation sensitivity of KYSE-150R cells. Silencing circRNA_100367 reduced the number of clones of KYSE-150R cells under the radiation dose of 0Gy and 6Gy, and decreased the survival fraction of KYSE-150R cells under the radiation dose of 4Gy, 6Gy and 8Gy (Figure 3B). In addition, the migration ability of KYSE-150R cells was stronger than KYSE-150 cells, and silencing circRNA_100367 decreased the migration of KYSE-150R cells (Figure 3C). Moreover, we found circRNA_100367 silence reduced the expression of β-catenin, Vimentin and Snail in KYSE-150R cells (Figure 3D).

### The interaction between circRNA_100367 and miR-217

**Figure 4:**
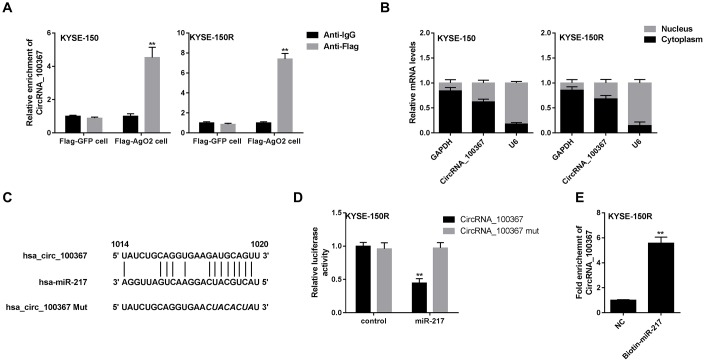
**The interaction between circRNA_100367 and miR-217.** (**A**) KYSE-150 and KYSE-150R cells were transfected with Flag-tagged AgO2 (Flag-AgO2) or negative control Flag-tagged Green Fluorescent Protein (Flag-GFP). Then cell lysates were incubated with Flag or IgG (negative control of Flag) antibodies. The enrichment of circRNA_100367 in the immunoprecipitation products was detected by qPCR. **p<0.01 vs. Anti-IgG. (**B**) The expression of circRNA_100367 in the nucleus and cytoplasm of KYSE-150 and KYSE-150R cells was detected by qRT-PCR. (**C**) The diagram showed the binding sites between hsa_circRNA_100367 and miR-217. (**D**) KYSE-150R cells were co-transfected with miR-217 mimic and luciferase reporter plasmids containing circRNA_100367 or circRNA_100367 mut. The relative luciferase activity was detected by dual luciferase reporter gene assay. **p<0.01 vs. circRNA_100367 mut. (**E**) The interaction between miR-217 and circRNA_100367 was assessed by RNA pull-down assay. **p<0.01 vs. NC.

Given that circRNAs can be acted as miRNA sponge and circRNA_100367 is abundant in the cytoplasm, we wonder if circRNA_100367 can bind to miR-217 to affect the anti-radioresistant phenotypes. We conducted immunoprecipitation and dual luciferase reporter gene assay to investigate whether circRNA_100367 could bind to miR-217. Anti-AgO2 immunoprecipitation showed circRNA_100367 was enriched in KYSE-150 and KYSE-150R cells treated with AgO2 antibody, suggesting the occupancy of AgO2 in the region of the circRNA_100367 (Figure 4A). Besides, we found most of circRNA_100367 are located in the cytoplasm of KYSE-150 and KYSE-150R cells (Figure 4B). Also, the binding sites between hsa_circRNA_100367 and miR-217 were analyzed by bioinformatics software (circinteractome) (Figure 4C). The luciferase activity of miR-217 was significantly increased in circRNA_100367 mut (Figure 4D), indicating miR-217 could bind to the binding sites of circRNA_100367. Moreover, the enrichment of circRNA_100367 in biotin-labeled miR-217 further proved the interaction between circRNA_100367 and miR-217 (Figure 4E).

### Wnt3 regulated the proliferation and migration of KYSE-150 cells

**Figure 5:**
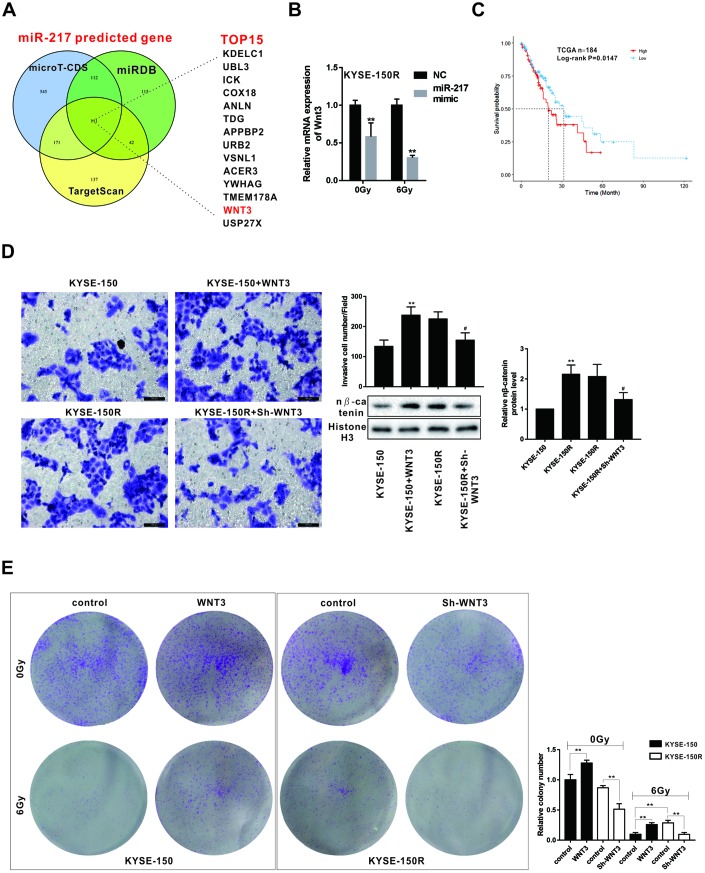
**Effects of Wnt3 on the proliferation and migration of KYSE-150 and KYSE-150R cells.** (**A**) The potential target genes of miR-217 were predicted by bioinformatics softwares (microT-CDS, miRDB and TargetScan). (**B**) KYSE-150R cells were transfected with miR-217 mimic (or NC) and then irradiated with 0 and 6 Gy X-ray. The mRNA level of Wnt3 was detected by qRT-PCR. **p<0.01 vs NC. (**C**) The relationship between Wnt3 expression and survival rate of patients with ESCS. (**D**) KYSE-150 cells were transfected with Wnt3 overexpression vector and KYSE-150R cells were transfected with Wnt3 silence vector. The invasion of KYSE-150 and KYSE-150R cells were detected by transwell assay, and the protein level of nuclear β-catenin (n β-catenin) was determined by western blot. **p<0.01 vs. KYSE-150; #p<0.05 vs. KYSE-150R. (**E**) KYSE-150 cells were transfected with Wnt3 overexpression vector and KYSE-150R cells were transfected with Wnt3 silence vector. Then cells were irradiated with 0 and 6 Gy X-ray. The proliferation of cells was evaluated by colony formation assay.

According to the bioinformatics software microT-CDS, miRDB and TargetScan, Wnt was one of the potential target genes of miR-217 with higher scores (Figure 5A). The other target genes of miR-217 were shown in the Supplementary Table 2. Wnt3 mRNA level was decreased in KYSE-150R cells after the treatment of miR-217 mimic under the radiation dose of 0Gy and 6Gy (Figure 5B). According to the data from The Cancer Genome Atlas (TCGA), there is an association between lower expression of Wnt3 and survival in patients with ESCC (n=184) (Figure 5C). As shown in Figure 5D, overexpression of Wnt3 promoted the migration of KYSE-150 cells, while knockdown of Wnt3 inhibited the migration of KYSE-150R cells. In addition, sh-Wnt3 reduced the number of migrated KYSE-150R cells (Figure 5D). Wnt3 also affected the expression of nucleus β-catenin. Wnt3 overexpression promoted nucleus β-catenin expression in KYSE-150 cells, and sh-Wnt3 down-regulated nucleus β-catenin expression in KYSE-150R cells (Figure 5D). Wnt3 overexpression increased the number of clones of KYSE-150 cells under the radiation dose of 0Gy and 6Gy, suggesting Wnt3 overexpression enhanced the radioresistant of KYSE-150 cells (Figure 5E). And Sh-Wnt3 inhibited the number of clones of KYSE-150R cells under the radiation dose of 0Gy and 6Gy, suggesting Sh-Wnt3 inhibited the radioresistant of KYSE-150R cells (Figure 5E).

### CircRNA_100367 coordinated with miRNA-217 regulated the radioresistant of KYSE-150R cells through Wnt3

**Figure 6:**
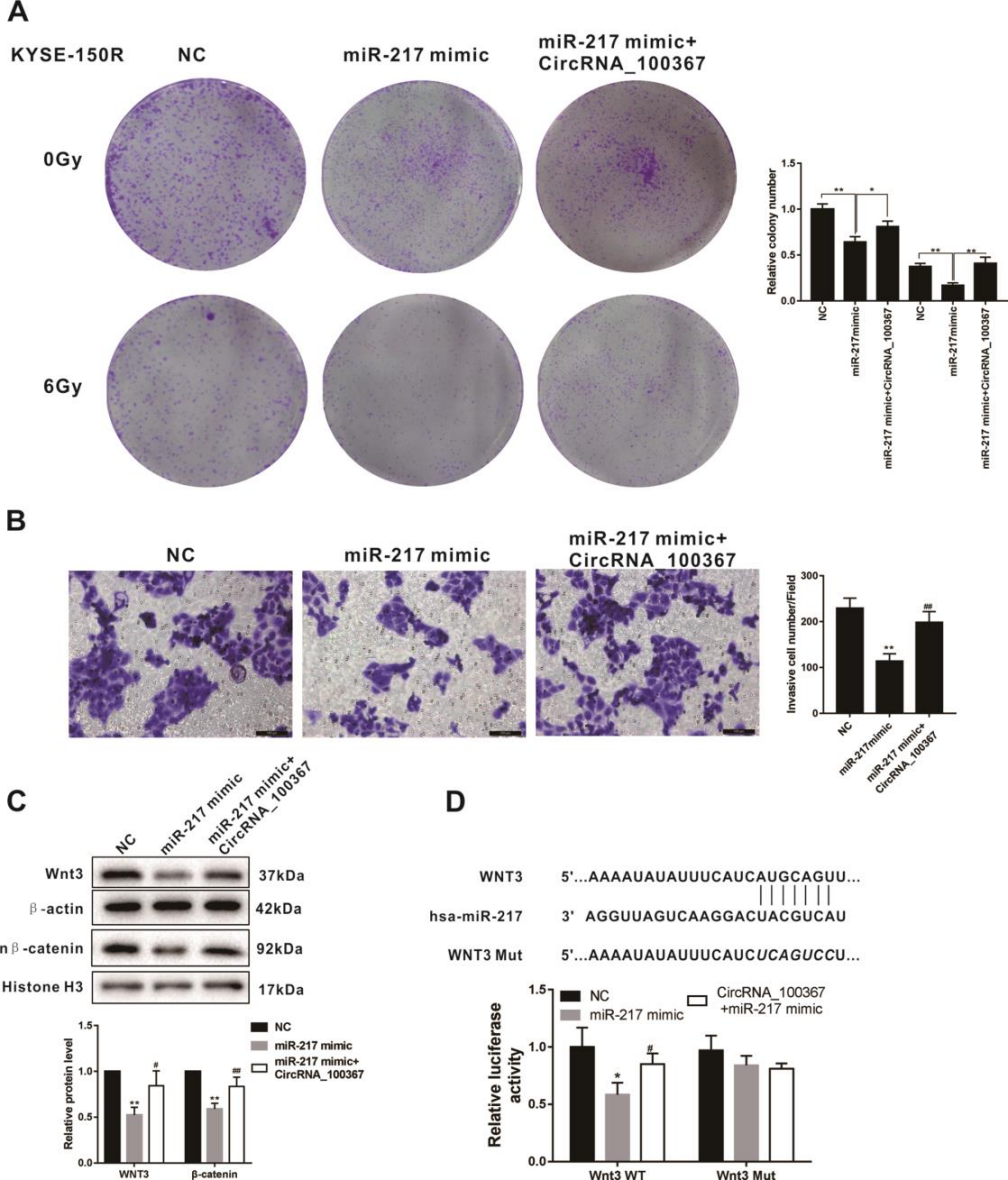
**CircRNA_100367 coordinated with miRNA-217 regulated the radioresistant of KYSE-150R cells through Wnt3.** (**A**) KYSE-150R cells were transfected with miR-217 mimic (or NC) or co-transfected with miR-217 mimic and circRNA_100367. Then cells were irradiated with 0 and 6 Gy X-ray. irradiated with 0 and 6 Gy X-ray. (**B**–**C**) KYSE-150R cells were transfected with miR-217 mimic (or NC) or co-transfected with miR-217 mimic and circRNA_100367. (**B**) The invasion of KYSE-150 and KYSE-150R cells were detected by transwell assay. (**C**) The protein levels of Wnt3 and nuclear β-catenin (n β-catenin) were detected by western blot. (**D**) KYSE-150R cells were co-transfected with miR-217 mimic, circRNA_100367 and luciferase reporter plasmids containing Wnt3 WT or Wnt3 mut. The relative luciferase activity was detected by dual luciferase reporter gene assay.

As shown in Figure 6A, miR-217 overexpression decreased the number of clones of KYSE-150R cells, whereas miR-217 mimic coordinated with circRNA_100367 increased the number of clones under the radiation dose of 0Gy and 6Gy. In addition, miR-217 overexpression reduced the number of KYSE-150R cells, whereas miR-217 mimic coordinated with circRNA_100367 increased the number of KYSE-150R cells (Figure 6B). miR-217 overexpression down-regulated the expressions of Wnt3 and nucleus β-catenin, whereas miR-217 mimic coordinated with circRNA_100367 up-regulated the expressions of Wnt3 and nucleus β-catenin (Figure 6C). Moreover, miRNA-217 overexpression decreased the luciferase activity of Wnt3 3’-UTR, whereas miRNA-217 mimic coordinated with circRNA_100367 increased the luciferase activity of Wnt3, indicating miRNA-217 and circRNA_100367 regulated cooperatively the 3′UTR of Wnt3 (Figure 6D).

### Silencing circRNA_100367 reduced the growth of KYSE-150R cells under radiation

**Figure 7:**
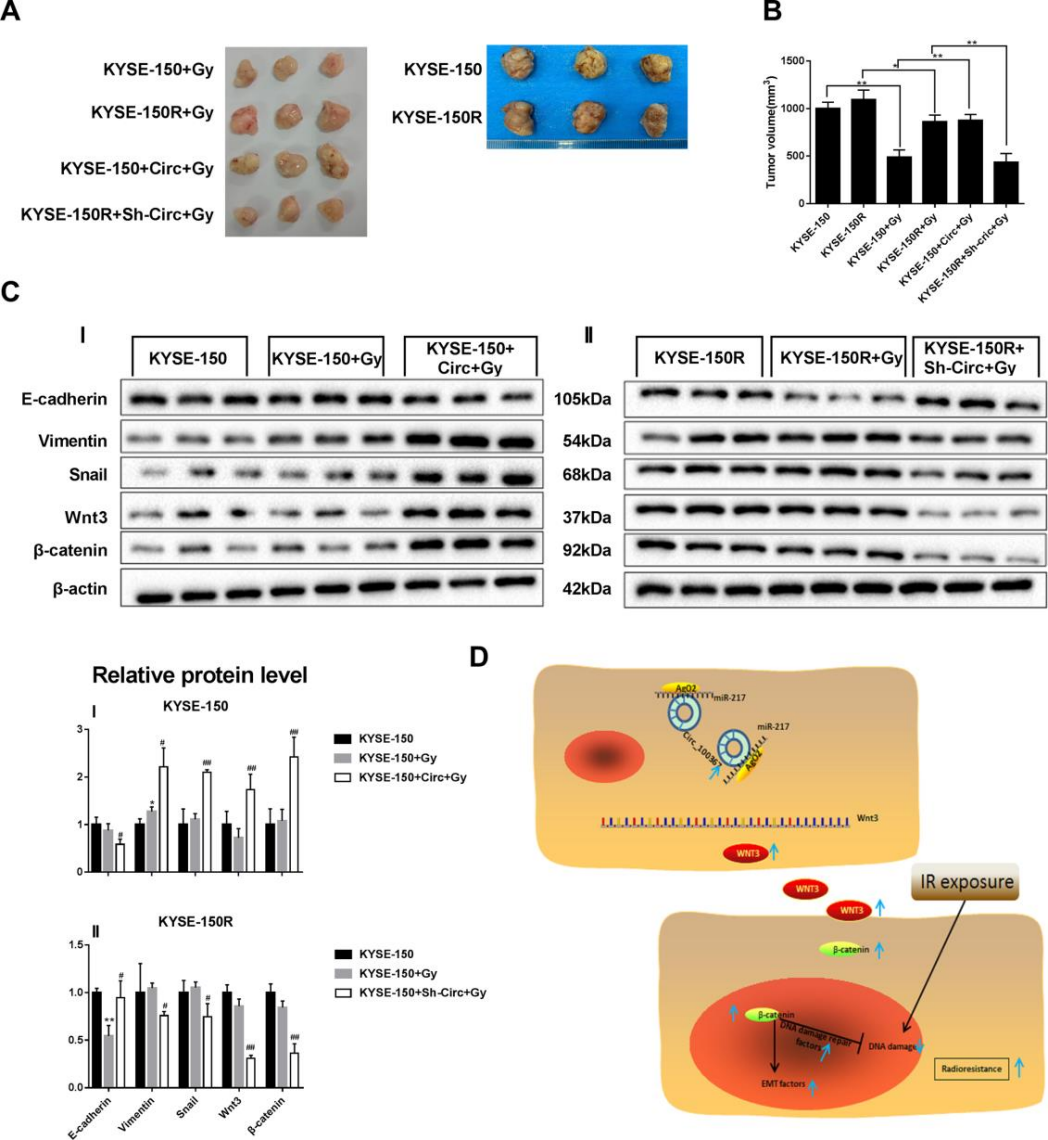
**Effect of circRNA_100367 on tumor growth of KYSE-150R cells under radiation.** KYSE-150R cells were stably transfected with Sh-circRNA_100367 or negative control (Circ) and then were subcutaneously inoculated into nude mice. 10 days after inoculation, mice were irradiated with 6 Gy X-ray. (**A**, **B**) Representative pictures and volumes of excised tumors. (**C**) The protein levels of E-cadherin, vimentin, snail, Wnt3 and β-catenin in excised tumors were measured by western blot. (**D**) A schematic diagram representing the role and mechanism of circRNA_100367 in radiation sensitivity of ESCC.

Since silencing circRNA_100367 inhibited the proliferation and migration of KYSE-150R cells *in vitro*, we determined the role of circRNA_100367 *in vivo* using a xenograft nude mouse model. KYSE-150 cells transfected with circRNA_100367 stably expressed high circRNA_100367 level, and KYSE-150R cells transfected with sh-circRNA_100367 stably expressed low circRNA_100367 level. We found that circRNA_100367 overexpression accelerated the growth of KYSE-150 cells, and silencing circRNA_100367 reduced the growth of KYSE-150R cells under radiation (Figure 7A). Tumor volume was significantly larger in KYSE-150R+Gy group than that of KYSE-150+Gy group. CircRNA_100367 overexpression significantly increased the tumor volume of KYSE-150+circRNA_100367+Gy group, and silencing circRNA_100367 significantly reduced the tumor volume of KYSE-150R+sh-circRNA_100367+Gy group (Figure 7B). The protein level of E-cadherin was decreased and the protein levels of vimentin, snail, Wnt3, andβ-catenin were increased in the KYSE-150+sh-circRNA_100367+Gy group compared with KYSE-150+ Gy group. Also, the protein level of E-cadherin was elevated and the protein levels of vimentin, snail, Wnt3, andβ-catenin were reduced in the KYSE-150R+sh-circRNA_100367+Gy group compared with KYSE-150R+ Gy group (Figure 7C).

## DISCUSSION

Increasing evidences have revealed that the abnormal expressions of circRNAs are related to the radiation sensitivity of cancers [9, 10]. However, few studies focus on the abnormally expressed circRNAs in regulating radiation sensitivity of ESCC. In this study, the upregulation of circRNA_100367 was observed in KYSE-150/KYSE-150R cells with a most extent than the other two ESCC cell lines and their radioresistant cells. Also, previous studies showed abnormally expressed circRNAs are related with the phenotypic change of cancer cells [25, 26]. So we further transfected sh-circRNA_100367 into KYSE-150R cells to determine whether circRNA_100367 changed the phenotype of ESCC radioresistant cells. Results showed that silencing circRNA_100367 decreased the viability and survival fraction of KYSE-150R cells, reduced the number of clones of KYSE-150R cells, and inhibited the migration of KYSE-150R cells under radiation. These results indicated that upregulation of circRNA_100367 suppressed the radiation sensitivity of radioresistant ESCC KYSE-150R.

Previous researches have reported that disease-specific miRNAs can be sponged by circRNAs in many cancers [26, 27]. miR-217 is one of these disease-specific miRNAs and exerts its functional role in a variety of cancers [28]. For example, abnormally expressed miR-217 enhanced the chemosensitivity of acute myeloid leukemia and cervical carcinoma [17, 29]. However, whether abnormally expressed miR-217 involved in regulating the radiation sensitivity of ESCC is not clear. Based on the mechanism of circRNAs sponging miRNAs in cancers and miR-217 predicted as a target of circRNA_100367, we conducted RIP and luciferase reporter gene assay, and proved the interaction between circRNA_100367 and miR-217. To investigate the in-depth underlying mechanism of circRNA_100367/miR-217, miR-217 mimic or miR-217 mimic+circRNA_100367 was transfected into KYSE-150R cells to determine their effect on colony formation and migration of KYSE-150R cells. Results showed miR-217 mimic coordinated with circRNA_100367 promoted colony formation and migration of KYSE-150R cells under the radiation dose, which indicated miR-217 mimic+circRNA_100367 attenuated radiation sensitivity of KYSE-150R cells. So far, no other studies have demonstrated the role of circRNA_100367/miR-217 in the regulation of radiation sensitivity of KYSE-150R cells, which will provide directions for the improving the survival rate of ESCC.

Wnt3, as a member of Wnt family, has been proved to promote the stabilization of β-catenin to regulate the radiation sensitivity of cancer cells [30, 31]. In this study, we found silencing Wnt3 down-regulated the expression of nucleus β-catenin, which was consistent with previous report [31]. However, the exact role of Wnt3 in the regulation of radiation sensitivity of ESCC cells is still unclear, although Wnt-β-catenin signaling has been reported as an important pathway in regulating the radioresistance of ESCC [32]. In this study, results showed that silencing Wnt3 enhanced the radiation sensitivity of KYSE-150R cells. In addition, silencing Wnt3 changed the phenotype of KYSE-150R cells, which suppressed the colony formation and the migration of KYSE-150R cells. These findings suggested that Wnt3 suppressed the radiation sensitivity of ESCC cells. Moreover, studies have demonstrated that Wnt3 can be the target of disease-specific miRNAs [33, 34]. In this study, we investigated the relationship between miR-217 and Wnt3 in KYSE-150R cells, and proved Wnt3 was a target of miR-217 according to the bioinformatics analysis and luciferase reporter gene assay. And Wnt3 expression was found to be negatively correlated with miR-217. Furthermore, circRNA_100367 reversed the effect of miR-217 on Wnt3 expression, the number of clones of KYSE-150R cells and the migration of KYSE-150R cells. These results indicated that miR-217/Wnt3 was the downstream targets of circRNA_100367 in regulating the radiation sensitivity of ESCC.

In conclusion, highly expressed circRNA_100367 is related with the radiation sensitivity of ESCC, and silencing circRNA_100367 decreased the proliferation and migration of KYSE-150R cells *in vitro*, and inhibited tumor growth *in vivo*. In addition, miR-217/Wnt3 was demonstrated as the downstream targets of circRNA_100367 in regulating the radiation sensitivity of ESCC (Figure 7D), which will provide potential targets for reducing radiotherapy failure of patients with ESCC.

## MATERIALS AND METHODS

### Cell culture and transfection

Esophageal cancer cell lines TE-1, KYSE-150 and ECA-109 were purchased from Cell Bank of Chinese Academy of Sciences, and cultured in RPMI-1640 (Gibco, Grand Island, NY, USA) supplemented with 10% fetal bovine serum (FBS; Gibco), 100 unit/ml of penicillin (Gibco) and 100 mg/ml of streptomycin (Gibco) at 37°C in an incubator containing 5% CO_2_ and 95% air.

Short hairpin RNA that targets human circRNA_100367 (sh-circRNA_100367), pcDNA3.0 vectors overexpressing circRNA_100367 (circRNA_100367), sh-Wnt3, and negative controls were synthesized by Genechem (Shanghai, China). miR-217 mimic (Cat. No. miR10000274-1-5) and its negative control were synthesized by RiboBio (Guangzhou, China). 100 nmol vectors were transfected into cells using Lipofectamine 2000 (Invitrogen, UAS). The sequences of those vector were as follows: miR-217 mimic 5′UACUGCAUCAGGAACUGAUUGGA3′; Sh-Wnt3 5′GCGCTTCTGCCGCAATTACAT3′; Sh-CircRNA-100367 5′GCGTCTCCTTCAGTGAATCTA3′; Sh-NC 5′TTCTCCGAACGTGTCACGTCT3′.

### Development of radioresistant esophageal cancer cells

Radioresistant esophageal cancer cell lines TE-1R, KYSE-150R and ECA-109R were established by multiple fractionated irradiation according to previous report [35]. TE-1, KYSE-150 and ECA-109 cells (1.5×10^6^) were seeded into 25 cm^2^ culture flasks, and irradiated with 1 Gy X-ray. Culture medium was immediately replaced after irradiation. After cells reached 90% confluence, cells were transferred to new culture flasks. Then, cells received second irradiation when they reached 50% confluence. In total, cells received 1Gy for three times, 2Gy for three times, 4Gy for three times with a total dose of 21Gy to establish radioresistant cells.

### Quantitative real-time PCR (qRT-PCR)

Total RNA was extracted from TE-1, KYSE-150 and ECA-109 cells using Trizol Reagent (Invitrogen, Carlsbad, CA, USA). The nucleus and cytoplasm were extracted by NE-PER™ Nuclear and Cytoplasmic Extraction Reagents (Thermo Scientific, Waltham, MA, USA). 1μg RNA was used to synthesize cDNA using PrimeScript II 1^st^ Strand cDNA Synthesis Kit (Takara Biomedical Technology, Beijing, China). qRT-PCR was performed with One Step TB Green PrimeScript PLUS RT-PCR Kit (Takara) using 7500 Real-Time PCR System (Applied Biosystems, FosterCity, CA, USA). GAPDH was used as the internal reference for circRNAs and Wnt3. The 2^-ΔΔCT^ [36] method was used to calculate the relative expressions of circRNA_100385, circRNA_104983, circRNA_001059, circRNA_100984, circRNA_100367, circRNA_103783, circRNA_100312 and Wnt3. The primer of U6 was purchased from Genechem (Cat. No. MQPS0000002-1-100; Shanghai, China). The sequences of other primers were shown in the Supplementary Table 1.

### Western blotting

KYSE-150 and KYSE-150R cells were collected and lysed using RIPA Lysis and Extraction Buffer (Thermo Scientific), and protein concentration was measured by BCA Protein Assay Kit (Beyotime Biotechnology, Shanghai, China). The protein samples were separated by 10% SDS-PAGE and transferred to PVDF membranes (Millipore, Billerica, MA, USA). The membrane was incubated with 5% skim milk at room temperature for 2 h, then incubated with primary antibodies against E-cadherin (1:10000; ab40772, Abcam), vimentin (1:2000; ab92547, Abcam), snail (1:200; ab82846, Abcam), β-catenin (1:5000; ab227499, Abcam), Wnt3 (1:1000; ab32249, Abcam), β-actin (1:5000; ab227387, Abcam), histone H3 (1:1000; MA5-15150, Abcam) overnight at 4°C. The membrane was subsequently incubated with HRP-conjugated secondary antibodies (1:5000) at room temperature for 1.5 h. The proteins were visualized using enhanced chemiluminescence (Thermo Scientific), and the band intensity was determined by ChemiDoc MP Imaging System (Bio-Rad, Hercules, CA, USA).

### Transwell assay

Transwell assay was conducted to detect the migration ability of KYSE-150 and KYSE-150R cells. Cells (2×10^4^ cells) were seeded on the upper chamber of Transwell, and RPMI-1640 medium with 10% FBS was added to the bottom chamber. After 48 h, cells on the bottom chamber were fixed with 4% paraformaldehyde and stained with 0.1% crystal violet solution and counted.

### Flow cytometry

KYSE-150 cells and KYSE-150R cells were stained with anti-CD133 antibody (Invitrogen) according to the manufacturer’s instruction. The percentage of CD133+ cells of KYSE-150 cells and KYSE-150R cells was determined by flow cytometry analysis using FACS Calibur (BD, San Jose, CA, USA).

### Colony formation assay

The 1.2% and 0.7% low-point agarose solutions were prepared at first. The 1:1 mixture of 1.2% agrose solution and RPMI-1640 medium was added into 6-well plates (1.5mL/well). The 0.7% agarose solution was mixed with RPMI-1640 medium supplemented with FBS, and KYSE-150 cells and KYSE-150R cells were resuspended in this mixture. Then cells (1×10^3^cells/ well) were seeded in 6-well plates and incubated for two weeks at 37°C. Then, colonies were fixed with 4% paraformaldehyde, stained with 0.1% crystal violet solution and counted.

### RNase R treatment

Total RNA (2 μg) was incubated with 3 U/μg RNase R (Epicentre Technologies, Madison, WI, USA) for 15 min at 37°C. Then, the RNA expression levels of DCAF8 and circRNA_100367 were detected by qRT-PCR.

### Cell proliferation assay

1-(4,5-Dimethylthiazol-2-yl)-3,5-diphenylformazan (MTT) assay kit (Sigma-Aldrich, Billerica, MA, USA) was used to detect the proliferation of KYSE-150 cells and KYSE-150R cells with different treatments under the radiation dose of 0Gy, 3Gy and 6Gy. Cells (2×10^3^ cells/well) were seeded into a 24-well plate, then 10 μl MTT solution was added to the cells in each well and incubated for 4 h at 37°C. The supernatant was discarded, then 200μl dimethyl sulfoxide (DMSO, Sigma-Aldrich) were added to the cells. The absorbance at the 490 nm was analyzed using a microplate reader.

### Wound healing assay

KYSE-150 cells and KYSE-150R cells with different treatments were seeded, and a linear wound was generated within the monolayers by scraping the cells using the sterile pipette tip. After 24 h of incubation, migration was imaged with an inverted microscope.

### RNA immunoprecipitation

Magna RIP RNA-Binding Protein Immunoprecipitation Kit (Millipore) was used to perform RNA immunoprecipitation (RIP) assay. The lysate of KYSE-150 cells or KYSE-150R cells was prepared by 1.5×10^7^ cells using 0.25 μl protease inhibitor, 0.125 μl RNase inhibitor and 50 μl RIP lysis buffer. After centrifugation, the supernatant was collected and incubated with RIP wash buffer, and the procedures were performed according to the manufacturer’s instruction. Finally, qRT-PCR was conducted to detect circRNA_100367 in RNA-binding protein complex absorbed by the magnetic beads.

### Dual luciferase reporter gene assay

According to the bioinformatics software (circinteractome), we predicted the binding sties between circRNA_100367 and miR-217. According to the bioinformatics software (microT-CDS, miRDB and TargetScan), we predicted the potential target genes of miR-217. CircRNA_100367 wild-type (circRNA_100367) and mutant (circRNA_100367 mut) reporter vectors were constructed and inserted into the psiCHECK-2 (Promega, Madison, Wisconsin, USA). Wnt3 wild-type (Wnt3 WT) and Wnt3 mutant (Wnt3 mut) reporter vectors were also constructed and inserted into the psiCHECK-2 (Promega). 100 nmol reporter plasmids (circRNA_100367, circRNA_100367 mut) together with miR-217 or negative control were transfected into KYSE-150R cells. The reporter plasmids (Wnt3 WT, Wnt3 mut) together with miR-217 mimic, circRNA_100367+miR-217 mimic, or negative control were transfected into KYSE-150R cells. Finally, Dual-Luciferase Reporter System Kit (Promega) was used to detect the luciferase activity.

### RNA pull-down

KYSE-150R cells (1.5×10^7^) were collected and lysed. The biotinylated miR-217 probe was synthesized by Genepharm (Shanghai, China) and used for incubation with streptavidin agarose beads (Thermo Scientific). Cell lysate with miR-217 probe or oligo probe was incubated for one night at 4°C. RNA complex bound to the beads was eluted by wash buffer, and qRT-PCR was used to measure circRNA_100367 enrichment pulled down by miR-217 probe.

### Xenografts experiments

This animal study was approved by the Ethics Committee of the First Affiliated Hospital of Zhengzhou University. KYSE-150 cells (5×10^6^) transfected with circRNA_100367 or KYSE-150R cells (5×10^6^) transfected with sh-circRNA_100367 were subcutaneously injected into the back of BALB/c female nude mice (6–8 weeks). All mice received radiotherapy 10 days after inoculation, with a radiation dose of 6Gy. So, the nude mice were divided into four groups, namely KYSE-150+Gy group (n=5), KYSE-150R+Gy group (n=5), KYSE-150+circRNA_100367+Gy group (n=5) and KYSE-150R+sh-circRNA_100367+Gy group (n=5). Tumor volume was measured 21 days later using the formula: tumor volume (mm3)=(length×width^2^)/2.

### Statistical analysis

The data were presented as mean±standard deviation (SD), and were analyzed using SPSS software (version 20; Chicago, IL, USA). Log-rank test was used to compare the survival rates between high-expression Wnt3 group and low-expression Wnt3 group by Kaplan-Meier analysis The significance of difference was detected using t test or one-way analysis of variance (ANOVA), with p<0.05 considered significant difference.

## Supplementary Material

Supplementary Tables

Supplementary Table 2
