# Correction for: CircRNA_100367 regulated the radiation sensitivity of esophageal squamous cell carcinomas through miR-217/Wnt3 pathway

**DOI:** 10.18632/aging.203664

**Published:** 2021-10-31

**Authors:** Junqi Liu, Nannan Xue, Yuexin Guo, Kerun Niu, Liang Gao, Song Zhang, Hao Gu, Xin Wang, Di Zhao, Ruitai Fan

**Affiliations:** 1 Department of Radiation Oncology, The First Affiliated Hospital of Zhengzhou University, Erqi, Zhengzhou, 450000, China; 2 Department of Molecular and Radiation Oncology, German Cancer Research Center (DKFZ), Heidelberg, 69120, Germany; 3 Center of Experimental Orthopaedics, Saarland University Medical Center, Kirrberger Strasse, Homburg, 66421, Germany; 4 Endocrinology Department, The First Affiliated Hospital of Zhengzhou University, Zhengzhou, 450000, China

**Keywords:** correction

Original article: Aging. 2019; 11:12412–12427.  . https://doi.org/10.18632/aging.102580

**This article has been corrected:** The authors recently found errors in **Figure 2B** and **5D** - the images for the "KYSE-150" group were incorrect. The authors corrected panels 2B and 5D in **Figure 2** and **Figure 5** by using representative images from the original sets of experiments. These alterations do not affect the results or conclusions of this work. The authors would like to apologize for any inconvenience caused.

New **Figures 2** and **5** are presented below.

**Figure 2:**
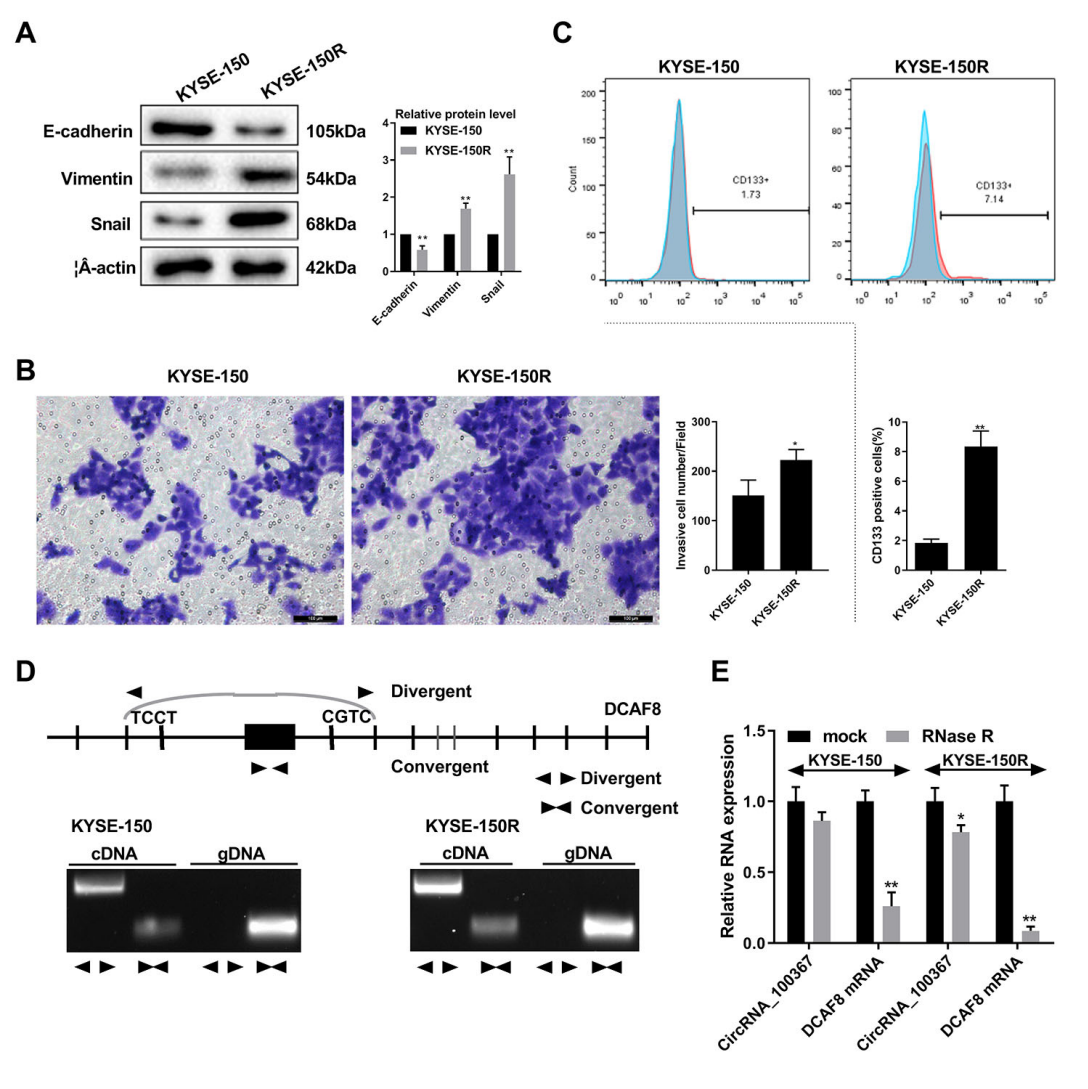
**CircRNA_100367 exists in KYSE-150R cells with higher potency of endothelial to mesenchymal transformation (EMT). **(**A**) The protein levels characteristic molecules (β-catenin, Vimentin, and Snail) of EMT in KYSE-150 and KYSE-150R cells were detected by western blot. (**B**) The migration of KYSE-150 and KYSE-150R cells were determined by transwell assay. (**C**) The CD133 positive cells of KYSE-150 and KYSE-150R cells were measured by flow cytometry. (**D**) Divergent primers and convergent primers were designed, and PCR product of cricRNA 100367 was detected in 1.5% agarose gel electrophoresis. (**E**) After the treatment of RNase R, circRNA_100367 and DCAF8 mRNA expressions in KYSE-150 and KYSE-150R cells were detected by qRT-PCR. *p<0.05, p<0.01 vs. KYSE-150 or mock.

**Figure 5:**
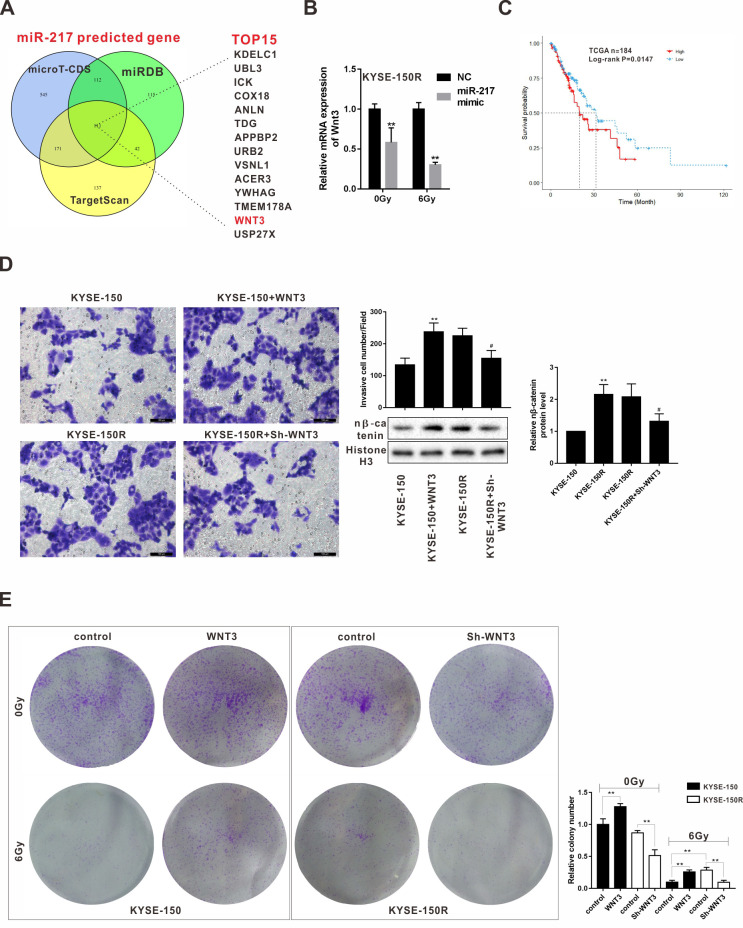
**Effects of Wnt3 on the proliferation and migration of KYSE-150 and KYSE-150R cells. **(**A**) The potential target genes of miR-217 were predicted by bioinformatics softwares (microT-CDS, miRDB and TargetScan). (**B**) KYSE-150R cells were transfected with miR-217 mimic (or NC) and then irradiated with 0 and 6 Gy X-ray. The mRNA level of Wnt3 was detected by qRT-PCR. **p<0.01 vs NC. (**C**) The relationship between Wnt3 expression and survival rate of patients with ESCS. (**D**) KYSE-150 cells were transfected with Wnt3 overexpression vector and KYSE-150R cells were transfected with Wnt3 silence vector. The invasion of KYSE-150 and KYSE-150R cells were detected by transwell assay, and the protein level of nuclear β-catenin (n β-catenin) was determined by western blot. **p<0.01 vs. KYSE-150; #p<0.05 vs. KYSE-150R. (**E**) KYSE-150 cells were transfected with Wnt3 overexpression vector and KYSE-150R cells were transfected with Wnt3 silence vector. Then cells were irradiated with 0 and 6 Gy X-ray. The proliferation of cells was evaluated by colony formation assay.

