# Supplementary material for: CircRNA_100367 regulated the radiation sensitivity of esophageal squamous cell carcinomas through miR-217/Wnt3 pathway
Source: Aging (Albany NY). 2019 Dec 18;11(24):12412–27. doi: 10.18632/aging.102580 (PMC6949088; doi:10.18632/aging.102580)
Supplement: Supplementary Tables [file aging-11-102580-s002..pdf]

SUPPLEMENTARY TABLES

Supplementary Table 1. Sequence information of primers.

Primers	Sequences (5′-3′)
CircRNA_100385	F: CAGGAGACGTGACTGCTGTG<br>R: TGTCCTATTCCTTCGCTGCT
CircRNA_104983	F: CCTTTCCATTGCAGCTCCT<br>R: CGCGGTAGTACACACTCAGC
CircRNA_001059	F: GGCACTAGGGAGGGACTCAT<br>R: GGGCAGAGACAGAGTGGATG
CircRNA_100984	F: CTGGCTTCAGAAAAGGATGC<br>R: TGAAAAGAGGGAGAGCTGGA
CircRNA_100367	F: GGCACTGGAACCAGACTCTC<br>R: GTAGCCAGCCTTTCCTCCTT
CircRNA_103783	F: CTGTTAGCATGATCCCACCAC<br>R: TTTTCCAAGTGTGGCGATTT
CircRNA_100312	F:TTGGCTGAGAAACTCCTTCC<br>R: GCTTCTTCCAAGGCCTTCTC
DCAF8	F: TGGCCTAGACCATGATGTGA<br>R: GTGATGGCGTCTCTGTCTCA
GADPH	F: AGAAGGCTGGGGCTCATTTG<br>R: AGGGGCCATCCACAGTCTTC
WNT3	F: ACGAGAACTCCCCCAACTTT<br>R: AGATGCAGTGGCATTTTTCC
Divergent primers	F: GGCACTGGAACCAGACTCTC<br>R: GTAGCCAGCCTTTCCTCCTT
Convergent primers	F: GAAGAGGGGAGGGAGACATC<br>R: TCTGTGTCTGTGCCTCGACT

Please browse Full Text version to see the data of Supplementary Table 2

Supplementary Table 2. Prodicted targets of miR-217.
