# Supplementary material for: CircRNA_100367 regulated the radiation sensitivity of esophageal squamous cell carcinomas through miR-217/Wnt3 pathway
Source: Aging (Albany NY). 2019 Dec 18;11(24):12412–27. doi: 10.18632/aging.102580 (PMC6949088; doi:10.18632/aging.102580)
Supplement: Supplementary Table 2 [file aging-11-102580-s001..docx]

Supplementary Table 2. Prodicted targets of miR-217.

| No. | microT-CDS | miRDB | TargetScan | common gene |
| --- | --- | --- | --- | --- |
| 1 | BCL11A | [FRMD5](http://www.ncbi.nlm.nih.gov/entrez/query.fcgi?db=gene&cmd=Retrieve&dopt=full_report&list_uids=84978) | KDELC1 | KDELC1 |
| 2 | TMTC4 | [ATP11C](http://www.ncbi.nlm.nih.gov/entrez/query.fcgi?db=gene&cmd=Retrieve&dopt=full_report&list_uids=286410) | UBL3 | UBL3 |
| 3 | PROX1 | [C5orf24](http://www.ncbi.nlm.nih.gov/entrez/query.fcgi?db=gene&cmd=Retrieve&dopt=full_report&list_uids=134553) | ZNF302 | ICK |
| 4 | SOS1 | [ACER3](http://www.ncbi.nlm.nih.gov/entrez/query.fcgi?db=gene&cmd=Retrieve&dopt=full_report&list_uids=55331) | PTP4A1 | COX18 |
| 5 | SUPT20H | [SCN9A](http://www.ncbi.nlm.nih.gov/entrez/query.fcgi?db=gene&cmd=Retrieve&dopt=full_report&list_uids=6335) | ICK | ANLN |
| 6 | CDYL2 | [MIER3](http://www.ncbi.nlm.nih.gov/entrez/query.fcgi?db=gene&cmd=Retrieve&dopt=full_report&list_uids=166968) | GMFB | TDG |
| 7 | RBFOX1 | [BCL11A](http://www.ncbi.nlm.nih.gov/entrez/query.fcgi?db=gene&cmd=Retrieve&dopt=full_report&list_uids=53335) | COX18 | APPBP2 |
| 8 | ANLN | [YTHDC1](http://www.ncbi.nlm.nih.gov/entrez/query.fcgi?db=gene&cmd=Retrieve&dopt=full_report&list_uids=91746) | ANLN | URB2 |
| 9 | SPOPL | [ATP1B1](http://www.ncbi.nlm.nih.gov/entrez/query.fcgi?db=gene&cmd=Retrieve&dopt=full_report&list_uids=481) | TDG | VSNL1 |
| 10 | LGMN | [ANLN](http://www.ncbi.nlm.nih.gov/entrez/query.fcgi?db=gene&cmd=Retrieve&dopt=full_report&list_uids=54443) | EIF1B | ACER3 |
| 11 | EZH2 | [RIMS2](http://www.ncbi.nlm.nih.gov/entrez/query.fcgi?db=gene&cmd=Retrieve&dopt=full_report&list_uids=9699) | APPBP2 | YWHAG |
| 12 | FRMD5 | [SLC38A2](http://www.ncbi.nlm.nih.gov/entrez/query.fcgi?db=gene&cmd=Retrieve&dopt=full_report&list_uids=54407) | URB2 | TMEM178A |
| 13 | PPM1D | [ATP8A1](http://www.ncbi.nlm.nih.gov/entrez/query.fcgi?db=gene&cmd=Retrieve&dopt=full_report&list_uids=10396) | VSNL1 | WNT3 |
| 14 | DACH1 | [TBC1D15](http://www.ncbi.nlm.nih.gov/entrez/query.fcgi?db=gene&cmd=Retrieve&dopt=full_report&list_uids=64786) | ACER3 | USP27X |
| 15 | ZSWIM8 | [UBL3](http://www.ncbi.nlm.nih.gov/entrez/query.fcgi?db=gene&cmd=Retrieve&dopt=full_report&list_uids=5412) | YWHAG | C5orf24 |
| 16 | ZNF92 | [PPM1D](http://www.ncbi.nlm.nih.gov/entrez/query.fcgi?db=gene&cmd=Retrieve&dopt=full_report&list_uids=8493) | CCNYL1 | SPOPL |
| 17 | ICK | [WNT3](http://www.ncbi.nlm.nih.gov/entrez/query.fcgi?db=gene&cmd=Retrieve&dopt=full_report&list_uids=7473) | TMEM178A | RABL3 |
| 18 | ATP8A1 | [BAI3](http://www.ncbi.nlm.nih.gov/entrez/query.fcgi?db=gene&cmd=Retrieve&dopt=full_report&list_uids=577) | EPT1 | DACH1 |
| 19 | TOPORS | [NAB1](http://www.ncbi.nlm.nih.gov/entrez/query.fcgi?db=gene&cmd=Retrieve&dopt=full_report&list_uids=4664) | WNT3 | TMTC4 |
| 20 | DACH2 | [DACH1](http://www.ncbi.nlm.nih.gov/entrez/query.fcgi?db=gene&cmd=Retrieve&dopt=full_report&list_uids=1602) | BEND6 | MIER3 |
| 21 | NIPBL | [EZH2](http://www.ncbi.nlm.nih.gov/entrez/query.fcgi?db=gene&cmd=Retrieve&dopt=full_report&list_uids=2146) | TCEB1 | OTUD7B |
| 22 | ATP1B1 | [FBN2](http://www.ncbi.nlm.nih.gov/entrez/query.fcgi?db=gene&cmd=Retrieve&dopt=full_report&list_uids=2201) | MPC1 | LMO7 |
| 23 | YTHDC1 | [TMTC4](http://www.ncbi.nlm.nih.gov/entrez/query.fcgi?db=gene&cmd=Retrieve&dopt=full_report&list_uids=84899) | USP27X | SEMA3A |
| 24 | SIRT1 | [ANK3](http://www.ncbi.nlm.nih.gov/entrez/query.fcgi?db=gene&cmd=Retrieve&dopt=full_report&list_uids=288) | DACH2 | ESCO1 |
| 25 | ZFYVE20 | [APPBP2](http://www.ncbi.nlm.nih.gov/entrez/query.fcgi?db=gene&cmd=Retrieve&dopt=full_report&list_uids=10513) | C5orf24 | WAPAL |
| 26 | ATP11C | [ICK](http://www.ncbi.nlm.nih.gov/entrez/query.fcgi?db=gene&cmd=Retrieve&dopt=full_report&list_uids=22858) | SPOPL | SLC4A5 |
| 27 | DDX53 | [WAPAL](http://www.ncbi.nlm.nih.gov/entrez/query.fcgi?db=gene&cmd=Retrieve&dopt=full_report&list_uids=23063) | RABL3 | FBXO11 |
| 28 | OTUD7B | [HIVEP3](http://www.ncbi.nlm.nih.gov/entrez/query.fcgi?db=gene&cmd=Retrieve&dopt=full_report&list_uids=59269) | DACH1 | CHN2 |
| 29 | C5orf24 | [EEA1](http://www.ncbi.nlm.nih.gov/entrez/query.fcgi?db=gene&cmd=Retrieve&dopt=full_report&list_uids=8411) | DMRT2 | HNF1B |
| 30 | FBN2 | [STT3A](http://www.ncbi.nlm.nih.gov/entrez/query.fcgi?db=gene&cmd=Retrieve&dopt=full_report&list_uids=3703) | C7orf41 | SIRT1 |
| 31 | WAPAL | [TACC2](http://www.ncbi.nlm.nih.gov/entrez/query.fcgi?db=gene&cmd=Retrieve&dopt=full_report&list_uids=10579) | TMTC4 | KRAS |
| 32 | CCNYL1 | [KCTD9](http://www.ncbi.nlm.nih.gov/entrez/query.fcgi?db=gene&cmd=Retrieve&dopt=full_report&list_uids=54793) | MAF | PPM1D |
| 33 | NFAT5 | [RAB4A](http://www.ncbi.nlm.nih.gov/entrez/query.fcgi?db=gene&cmd=Retrieve&dopt=full_report&list_uids=5867) | MIER3 | PALM2-AKAP2 |
| 34 | PKP4 | [PTPN21](http://www.ncbi.nlm.nih.gov/entrez/query.fcgi?db=gene&cmd=Retrieve&dopt=full_report&list_uids=11099) | AAK1 | STT3A |
| 35 | GPM6A | [DCBLD2](http://www.ncbi.nlm.nih.gov/entrez/query.fcgi?db=gene&cmd=Retrieve&dopt=full_report&list_uids=131566) | OTUD7B | RBFOX1 |
| 36 | C16orf52 | [SPOPL](http://www.ncbi.nlm.nih.gov/entrez/query.fcgi?db=gene&cmd=Retrieve&dopt=full_report&list_uids=339745) | PNRC2 | DCP2 |
| 37 | SNX4 | [LPAR1](http://www.ncbi.nlm.nih.gov/entrez/query.fcgi?db=gene&cmd=Retrieve&dopt=full_report&list_uids=1902) | YME1L1 | CERS6 |
| 38 | MIER3 | [GPM6A](http://www.ncbi.nlm.nih.gov/entrez/query.fcgi?db=gene&cmd=Retrieve&dopt=full_report&list_uids=2823) | LMO7 | ATP8A1 |
| 39 | TET1 | [NPAS3](http://www.ncbi.nlm.nih.gov/entrez/query.fcgi?db=gene&cmd=Retrieve&dopt=full_report&list_uids=64067) | GXYLT1 | BAI3 |
| 40 | LPPR4 | [PTPN14](http://www.ncbi.nlm.nih.gov/entrez/query.fcgi?db=gene&cmd=Retrieve&dopt=full_report&list_uids=5784) | SEMA3A | AGFG1 |
| 41 | EPT1 | [ASAP1](http://www.ncbi.nlm.nih.gov/entrez/query.fcgi?db=gene&cmd=Retrieve&dopt=full_report&list_uids=50807) | ESCO1 | DNAJC5 |
| 42 | SLC15A2 | [PPM1A](http://www.ncbi.nlm.nih.gov/entrez/query.fcgi?db=gene&cmd=Retrieve&dopt=full_report&list_uids=5494) | PPP4R2 | RTF1 |
| 43 | FIGN | [MAST4](http://www.ncbi.nlm.nih.gov/entrez/query.fcgi?db=gene&cmd=Retrieve&dopt=full_report&list_uids=375449) | ARID2 | GPM6A |
| 44 | HNF1B | [ZCCHC2](http://www.ncbi.nlm.nih.gov/entrez/query.fcgi?db=gene&cmd=Retrieve&dopt=full_report&list_uids=54877) | WAPAL | TM9SF3 |
| 45 | APPBP2 | [HECTD2](http://www.ncbi.nlm.nih.gov/entrez/query.fcgi?db=gene&cmd=Retrieve&dopt=full_report&list_uids=143279) | SLC4A5 | SENP7 |
| 46 | GNL1 | [YAF2](http://www.ncbi.nlm.nih.gov/entrez/query.fcgi?db=gene&cmd=Retrieve&dopt=full_report&list_uids=10138) | PROX1 | HNRNPA3 |
| 47 | CTDSPL2 | [HOMER1](http://www.ncbi.nlm.nih.gov/entrez/query.fcgi?db=gene&cmd=Retrieve&dopt=full_report&list_uids=9456) | CACHD1 | NPAS3 |
| 48 | UQCC1 | [DOCK4](http://www.ncbi.nlm.nih.gov/entrez/query.fcgi?db=gene&cmd=Retrieve&dopt=full_report&list_uids=9732) | FBXO11 | YAF2 |
| 49 | USP27X | [KLHL29](http://www.ncbi.nlm.nih.gov/entrez/query.fcgi?db=gene&cmd=Retrieve&dopt=full_report&list_uids=114818) | CHN2 | RAP2C |
| 50 | NCR3LG1 | [TDG](http://www.ncbi.nlm.nih.gov/entrez/query.fcgi?db=gene&cmd=Retrieve&dopt=full_report&list_uids=6996) | CUL4B | FBN2 |
| 51 | ZNF721 | [MORF4L2](http://www.ncbi.nlm.nih.gov/entrez/query.fcgi?db=gene&cmd=Retrieve&dopt=full_report&list_uids=9643) | HNF1B | FEM1C |
| 52 | SENP7 | [TM9SF3](http://www.ncbi.nlm.nih.gov/entrez/query.fcgi?db=gene&cmd=Retrieve&dopt=full_report&list_uids=56889) | MAPK1 | SEC23IP |
| 53 | TMEM67 | [RBFOX1](http://www.ncbi.nlm.nih.gov/entrez/query.fcgi?db=gene&cmd=Retrieve&dopt=full_report&list_uids=54715) | SIRT1 | ZNF711 |
| 54 | DOCK4 | [CHN2](http://www.ncbi.nlm.nih.gov/entrez/query.fcgi?db=gene&cmd=Retrieve&dopt=full_report&list_uids=1124) | KRAS | TBC1D15 |
| 55 | C19orf82 | [SAP30L](http://www.ncbi.nlm.nih.gov/entrez/query.fcgi?db=gene&cmd=Retrieve&dopt=full_report&list_uids=79685) | C18orf42 | YTHDC1 |
| 56 | PARD3B | [PCNA](http://www.ncbi.nlm.nih.gov/entrez/query.fcgi?db=gene&cmd=Retrieve&dopt=full_report&list_uids=5111) | PPM1D | STX1A |
| 57 | CHN2 | [FOXO3](http://www.ncbi.nlm.nih.gov/entrez/query.fcgi?db=gene&cmd=Retrieve&dopt=full_report&list_uids=2309) | PALM2-AKAP2 | RAB4A |
| 58 | PSMG1 | [ZNF711](http://www.ncbi.nlm.nih.gov/entrez/query.fcgi?db=gene&cmd=Retrieve&dopt=full_report&list_uids=7552) | STT3A | BRMS1L |
| 59 | KDELC1 | [FN1](http://www.ncbi.nlm.nih.gov/entrez/query.fcgi?db=gene&cmd=Retrieve&dopt=full_report&list_uids=2335) | MMGT1 | RIMS2 |
| 60 | URB2 | [SEMA3A](http://www.ncbi.nlm.nih.gov/entrez/query.fcgi?db=gene&cmd=Retrieve&dopt=full_report&list_uids=10371) | RBFOX1 | KCTD9 |
| 61 | NAB1 | [KIF2A](http://www.ncbi.nlm.nih.gov/entrez/query.fcgi?db=gene&cmd=Retrieve&dopt=full_report&list_uids=3796) | DCP2 | SOS1 |
| 62 | CUL5 | [SH3PXD2A](http://www.ncbi.nlm.nih.gov/entrez/query.fcgi?db=gene&cmd=Retrieve&dopt=full_report&list_uids=9644) | CERS6 | RUNX2 |
| 63 | COX18 | [MYEF2](http://www.ncbi.nlm.nih.gov/entrez/query.fcgi?db=gene&cmd=Retrieve&dopt=full_report&list_uids=50804) | ATP8A1 | TMEM135 |
| 64 | CLOCK | [ANO3](http://www.ncbi.nlm.nih.gov/entrez/query.fcgi?db=gene&cmd=Retrieve&dopt=full_report&list_uids=63982) | HMGN3 | CLIC4 |
| 65 | ATG7 | [HNF1B](http://www.ncbi.nlm.nih.gov/entrez/query.fcgi?db=gene&cmd=Retrieve&dopt=full_report&list_uids=6928) | BAI3 | FRMD5 |
| 66 | DNA2 | [LCA5](http://www.ncbi.nlm.nih.gov/entrez/query.fcgi?db=gene&cmd=Retrieve&dopt=full_report&list_uids=167691) | GKAP1 | CHST11 |
| 67 | MEF2C | [CHST11](http://www.ncbi.nlm.nih.gov/entrez/query.fcgi?db=gene&cmd=Retrieve&dopt=full_report&list_uids=50515) | NUPL1 | HOMER2 |
| 68 | LCOR | [SEC23IP](http://www.ncbi.nlm.nih.gov/entrez/query.fcgi?db=gene&cmd=Retrieve&dopt=full_report&list_uids=11196) | TMSB4X | MXI1 |
| 69 | ITPA | [ESCO1](http://www.ncbi.nlm.nih.gov/entrez/query.fcgi?db=gene&cmd=Retrieve&dopt=full_report&list_uids=114799) | AGFG1 | ADSS |
| 70 | SRSF10 | [SIRT1](http://www.ncbi.nlm.nih.gov/entrez/query.fcgi?db=gene&cmd=Retrieve&dopt=full_report&list_uids=23411) | DNAJC5 | SLC38A2 |
| 71 | SYCE1 | [HNRNPA3](http://www.ncbi.nlm.nih.gov/entrez/query.fcgi?db=gene&cmd=Retrieve&dopt=full_report&list_uids=220988) | INSIG2 | KLHL29 |
| 72 | GSTM2 | [YOD1](http://www.ncbi.nlm.nih.gov/entrez/query.fcgi?db=gene&cmd=Retrieve&dopt=full_report&list_uids=55432) | RTF1 | ANO3 |
| 73 | GMPS | [WDR48](http://www.ncbi.nlm.nih.gov/entrez/query.fcgi?db=gene&cmd=Retrieve&dopt=full_report&list_uids=57599) | ENAH | CHRDL1 |
| 74 | MPDZ | [PROSER1](http://www.ncbi.nlm.nih.gov/entrez/query.fcgi?db=gene&cmd=Retrieve&dopt=full_report&list_uids=80209) | GPM6A | SLC19A2 |
| 75 | DMRT2 | [EHMT1](http://www.ncbi.nlm.nih.gov/entrez/query.fcgi?db=gene&cmd=Retrieve&dopt=full_report&list_uids=79813) | TMEM66 | PDCD6IP |
| 76 | EPC1 | [NOVA1](http://www.ncbi.nlm.nih.gov/entrez/query.fcgi?db=gene&cmd=Retrieve&dopt=full_report&list_uids=4857) | DKK1 | REST |
| 77 | C11orf65 | [GXYLT1](http://www.ncbi.nlm.nih.gov/entrez/query.fcgi?db=gene&cmd=Retrieve&dopt=full_report&list_uids=283464) | TM9SF3 | RILPL1 |
| 78 | GDI2 | [TDO2](http://www.ncbi.nlm.nih.gov/entrez/query.fcgi?db=gene&cmd=Retrieve&dopt=full_report&list_uids=6999) | SENP7 | MYEF2 |
| 79 | ZNF711 | [PGM2L1](http://www.ncbi.nlm.nih.gov/entrez/query.fcgi?db=gene&cmd=Retrieve&dopt=full_report&list_uids=283209) | HNRNPA3 | LUC7L3 |
| 80 | TG | [RABL3](http://www.ncbi.nlm.nih.gov/entrez/query.fcgi?db=gene&cmd=Retrieve&dopt=full_report&list_uids=285282) | NPAS3 | LCOR |
| 81 | DNAJC5 | [NUFIP2](http://www.ncbi.nlm.nih.gov/entrez/query.fcgi?db=gene&cmd=Retrieve&dopt=full_report&list_uids=57532) | TACC2 | DOCK4 |
| 82 | FAM91A1 | [POLD3](http://www.ncbi.nlm.nih.gov/entrez/query.fcgi?db=gene&cmd=Retrieve&dopt=full_report&list_uids=10714) | YAF2 | TTC28 |
| 83 | FOXO3 | [ATF1](http://www.ncbi.nlm.nih.gov/entrez/query.fcgi?db=gene&cmd=Retrieve&dopt=full_report&list_uids=466) | RAP2C | CUL5 |
| 84 | GRSF1 | [ZFYVE20](http://www.ncbi.nlm.nih.gov/entrez/query.fcgi?db=gene&cmd=Retrieve&dopt=full_report&list_uids=64145) | MRPS28 | FOXO3 |
| 85 | TMEM178A | [KDELC1](http://www.ncbi.nlm.nih.gov/entrez/query.fcgi?db=gene&cmd=Retrieve&dopt=full_report&list_uids=79070) | FBN2 | ST8SIA3 |
| 86 | MORF4L2 | [PIKFYVE](http://www.ncbi.nlm.nih.gov/entrez/query.fcgi?db=gene&cmd=Retrieve&dopt=full_report&list_uids=200576) | CTDSPL2 | SH3PXD2A |
| 87 | RAP2C | [ADSS](http://www.ncbi.nlm.nih.gov/entrez/query.fcgi?db=gene&cmd=Retrieve&dopt=full_report&list_uids=159) | FEM1C | WWC3 |
| 88 | SLC16A10 | [NUPL1](http://www.ncbi.nlm.nih.gov/entrez/query.fcgi?db=gene&cmd=Retrieve&dopt=full_report&list_uids=9818) | SEC23IP | ZC3H4 |
| 89 | SH3PXD2A | [C14orf37](http://www.ncbi.nlm.nih.gov/entrez/query.fcgi?db=gene&cmd=Retrieve&dopt=full_report&list_uids=145407) | ZNF711 | PSMF1 |
| 90 | AGFG1 | [RAB9B](http://www.ncbi.nlm.nih.gov/entrez/query.fcgi?db=gene&cmd=Retrieve&dopt=full_report&list_uids=51209) | C10orf107 | RAPGEF6 |
| 91 | ZNF736 | [DACT1](http://www.ncbi.nlm.nih.gov/entrez/query.fcgi?db=gene&cmd=Retrieve&dopt=full_report&list_uids=51339) | GATSL2 | SLC39A10 |
| 92 | DLGAP2 | [KCNH5](http://www.ncbi.nlm.nih.gov/entrez/query.fcgi?db=gene&cmd=Retrieve&dopt=full_report&list_uids=27133) | TBC1D15 | FSD1L |
| 93 | TSPAN19 | [NFAT5](http://www.ncbi.nlm.nih.gov/entrez/query.fcgi?db=gene&cmd=Retrieve&dopt=full_report&list_uids=10725) | TBX20 | KCNH5 |
| 94 | ZNF773 | [GATAD2A](http://www.ncbi.nlm.nih.gov/entrez/query.fcgi?db=gene&cmd=Retrieve&dopt=full_report&list_uids=54815) | HERPUD1 | NIPBL |
| 95 | YWHAG | [KIAA2026](http://www.ncbi.nlm.nih.gov/entrez/query.fcgi?db=gene&cmd=Retrieve&dopt=full_report&list_uids=158358) | FGFR2 | ATP1B1 |
| 96 | TDG | [SLC31A1](http://www.ncbi.nlm.nih.gov/entrez/query.fcgi?db=gene&cmd=Retrieve&dopt=full_report&list_uids=1317) | ZNF207 | NOVA1 |
| 97 | TRIQK | [ZBTB24](http://www.ncbi.nlm.nih.gov/entrez/query.fcgi?db=gene&cmd=Retrieve&dopt=full_report&list_uids=9841) | YTHDC1 | DST |
| 98 | PTPN14 | [USP27X](http://www.ncbi.nlm.nih.gov/entrez/query.fcgi?db=gene&cmd=Retrieve&dopt=full_report&list_uids=389856) | STX1A | PIKFYVE |
| 99 | MPC1 | [URB2](http://www.ncbi.nlm.nih.gov/entrez/query.fcgi?db=gene&cmd=Retrieve&dopt=full_report&list_uids=9816) | RAB4A | ATP6V1A |
| 100 | ANTXR1 | [CPSF2](http://www.ncbi.nlm.nih.gov/entrez/query.fcgi?db=gene&cmd=Retrieve&dopt=full_report&list_uids=53981) | ZC3H12B | ANK3 |
| 101 | ZNF354B | [EXTL2](http://www.ncbi.nlm.nih.gov/entrez/query.fcgi?db=gene&cmd=Retrieve&dopt=full_report&list_uids=2135) | BRMS1L | HIVEP3 |
| 102 | HMOX1 | [RTF1](http://www.ncbi.nlm.nih.gov/entrez/query.fcgi?db=gene&cmd=Retrieve&dopt=full_report&list_uids=23168) | EDIL3 | SUZ12 |
| 103 | ANK3 | [CUL5](http://www.ncbi.nlm.nih.gov/entrez/query.fcgi?db=gene&cmd=Retrieve&dopt=full_report&list_uids=8065) | RIMS2 | CADPS2 |
| 104 | GOLGA6L4 | [ZNF438](http://www.ncbi.nlm.nih.gov/entrez/query.fcgi?db=gene&cmd=Retrieve&dopt=full_report&list_uids=220929) | FOXJ3 | MFSD6 |
| 105 | PTP4A1 | [IMPA1](http://www.ncbi.nlm.nih.gov/entrez/query.fcgi?db=gene&cmd=Retrieve&dopt=full_report&list_uids=3612) | TPD52L2 | MRE11A |
| 106 | MAPK1 | [LMLN](http://www.ncbi.nlm.nih.gov/entrez/query.fcgi?db=gene&cmd=Retrieve&dopt=full_report&list_uids=89782) | EHMT1 | ASAP2 |
| 107 | NAP1L2 | [TTC28](http://www.ncbi.nlm.nih.gov/entrez/query.fcgi?db=gene&cmd=Retrieve&dopt=full_report&list_uids=23331) | KCTD9 | USP37 |
| 108 | PHF6 | [HOMER2](http://www.ncbi.nlm.nih.gov/entrez/query.fcgi?db=gene&cmd=Retrieve&dopt=full_report&list_uids=9455) | ASB9 | FNDC3B |
| 109 | MAGI2 | [FAXC](http://www.ncbi.nlm.nih.gov/entrez/query.fcgi?db=gene&cmd=Retrieve&dopt=full_report&list_uids=84553) | OTUD1 | NUFIP2 |
| 110 | ZC3H4 | [RUNX2](http://www.ncbi.nlm.nih.gov/entrez/query.fcgi?db=gene&cmd=Retrieve&dopt=full_report&list_uids=860) | SOS1 | PDS5B |
| 111 | USP7 | [NR4A2](http://www.ncbi.nlm.nih.gov/entrez/query.fcgi?db=gene&cmd=Retrieve&dopt=full_report&list_uids=4929) | STAU2 | ATG7 |
| 112 | VSNL1 | [PPP4R2](http://www.ncbi.nlm.nih.gov/entrez/query.fcgi?db=gene&cmd=Retrieve&dopt=full_report&list_uids=151987) | SFPQ |  |
| 113 | INHBA | [RIN2](http://www.ncbi.nlm.nih.gov/entrez/query.fcgi?db=gene&cmd=Retrieve&dopt=full_report&list_uids=54453) | RUNX2 |  |
| 114 | GREB1L | [RAPGEF6](http://www.ncbi.nlm.nih.gov/entrez/query.fcgi?db=gene&cmd=Retrieve&dopt=full_report&list_uids=51735) | SENP5 |  |
| 115 | COL8A1 | [FNDC3B](http://www.ncbi.nlm.nih.gov/entrez/query.fcgi?db=gene&cmd=Retrieve&dopt=full_report&list_uids=64778) | RAB37 |  |
| 116 | FGFR2 | [FAR2](http://www.ncbi.nlm.nih.gov/entrez/query.fcgi?db=gene&cmd=Retrieve&dopt=full_report&list_uids=55711) | TMEM135 |  |
| 117 | ZNF667 | [NEXN](http://www.ncbi.nlm.nih.gov/entrez/query.fcgi?db=gene&cmd=Retrieve&dopt=full_report&list_uids=91624) | LYRM9 |  |
| 118 | LMO7 | [MTUS1](http://www.ncbi.nlm.nih.gov/entrez/query.fcgi?db=gene&cmd=Retrieve&dopt=full_report&list_uids=57509) | CDYL2 |  |
| 119 | RUNX2 | [PPP2R5E](http://www.ncbi.nlm.nih.gov/entrez/query.fcgi?db=gene&cmd=Retrieve&dopt=full_report&list_uids=5529) | SEL1L |  |
| 120 | ASAP2 | [STEAP3](http://www.ncbi.nlm.nih.gov/entrez/query.fcgi?db=gene&cmd=Retrieve&dopt=full_report&list_uids=55240) | SELK |  |
| 121 | PSIP1 | [TCEB1](http://www.ncbi.nlm.nih.gov/entrez/query.fcgi?db=gene&cmd=Retrieve&dopt=full_report&list_uids=6921) | NAP1L1 |  |
| 122 | ZNF714 | [C21orf62](http://www.ncbi.nlm.nih.gov/entrez/query.fcgi?db=gene&cmd=Retrieve&dopt=full_report&list_uids=56245) | USP15 |  |
| 123 | SEMA3A | [LIN9](http://www.ncbi.nlm.nih.gov/entrez/query.fcgi?db=gene&cmd=Retrieve&dopt=full_report&list_uids=286826) | DAB1 |  |
| 124 | HIPK3 | [EPHA5](http://www.ncbi.nlm.nih.gov/entrez/query.fcgi?db=gene&cmd=Retrieve&dopt=full_report&list_uids=2044) | CLIC4 |  |
| 125 | NICN1 | [YWHAG](http://www.ncbi.nlm.nih.gov/entrez/query.fcgi?db=gene&cmd=Retrieve&dopt=full_report&list_uids=7532) | FRMD5 |  |
| 126 | ZNF669 | [GBE1](http://www.ncbi.nlm.nih.gov/entrez/query.fcgi?db=gene&cmd=Retrieve&dopt=full_report&list_uids=2632) | INTS2 |  |
| 127 | HMGA2 | [ARL4A](http://www.ncbi.nlm.nih.gov/entrez/query.fcgi?db=gene&cmd=Retrieve&dopt=full_report&list_uids=10124) | CHST11 |  |
| 128 | RC3H2 | [AGGF1](http://www.ncbi.nlm.nih.gov/entrez/query.fcgi?db=gene&cmd=Retrieve&dopt=full_report&list_uids=55109) | HOMER2 |  |
| 129 | EIF4E2 | [ATG2B](http://www.ncbi.nlm.nih.gov/entrez/query.fcgi?db=gene&cmd=Retrieve&dopt=full_report&list_uids=55102) | MXI1 |  |
| 130 | NT5C1B-RDH14 | [RMND5A](http://www.ncbi.nlm.nih.gov/entrez/query.fcgi?db=gene&cmd=Retrieve&dopt=full_report&list_uids=64795) | NR4A2 |  |
| 131 | NDUFS4 | [NT5DC1](http://www.ncbi.nlm.nih.gov/entrez/query.fcgi?db=gene&cmd=Retrieve&dopt=full_report&list_uids=221294) | VAT1L |  |
| 132 | HSPA9 | [FEM1C](http://www.ncbi.nlm.nih.gov/entrez/query.fcgi?db=gene&cmd=Retrieve&dopt=full_report&list_uids=56929) | NF1 |  |
| 133 | ENAH | [TLR4](http://www.ncbi.nlm.nih.gov/entrez/query.fcgi?db=gene&cmd=Retrieve&dopt=full_report&list_uids=7099) | ADSS |  |
| 134 | TTC28 | [RILPL1](http://www.ncbi.nlm.nih.gov/entrez/query.fcgi?db=gene&cmd=Retrieve&dopt=full_report&list_uids=353116) | C5orf15 |  |
| 135 | ZBTB37 | [MAP1B](http://www.ncbi.nlm.nih.gov/entrez/query.fcgi?db=gene&cmd=Retrieve&dopt=full_report&list_uids=4131) | ZBTB21 |  |
| 136 | CADPS2 | [PANK1](http://www.ncbi.nlm.nih.gov/entrez/query.fcgi?db=gene&cmd=Retrieve&dopt=full_report&list_uids=53354) | NME6 |  |
| 137 | SURF4 | [CREB5](http://www.ncbi.nlm.nih.gov/entrez/query.fcgi?db=gene&cmd=Retrieve&dopt=full_report&list_uids=9586) | ZNF385D |  |
| 138 | PLXNA4 | [PSMF1](http://www.ncbi.nlm.nih.gov/entrez/query.fcgi?db=gene&cmd=Retrieve&dopt=full_report&list_uids=9491) | SLC38A2 |  |
| 139 | CCSER2 | [FAM169A](http://www.ncbi.nlm.nih.gov/entrez/query.fcgi?db=gene&cmd=Retrieve&dopt=full_report&list_uids=26049) | PLXNA4 |  |
| 140 | CHST11 | [ZNF799](http://www.ncbi.nlm.nih.gov/entrez/query.fcgi?db=gene&cmd=Retrieve&dopt=full_report&list_uids=90576) | AKT3 |  |
| 141 | ZNF799 | [ST8SIA3](http://www.ncbi.nlm.nih.gov/entrez/query.fcgi?db=gene&cmd=Retrieve&dopt=full_report&list_uids=51046) | FIGN |  |
| 142 | PCDH15 | [IARS](http://www.ncbi.nlm.nih.gov/entrez/query.fcgi?db=gene&cmd=Retrieve&dopt=full_report&list_uids=3376) | C4orf46 |  |
| 143 | USP15 | [GTF2H2C_2](http://www.ncbi.nlm.nih.gov/entrez/query.fcgi?db=gene&cmd=Retrieve&dopt=full_report&list_uids=730394) | KLHL29 |  |
| 144 | ARL5A | [NSUN3](http://www.ncbi.nlm.nih.gov/entrez/query.fcgi?db=gene&cmd=Retrieve&dopt=full_report&list_uids=63899) | GMPS |  |
| 145 | SLC26A5 | [TET2](http://www.ncbi.nlm.nih.gov/entrez/query.fcgi?db=gene&cmd=Retrieve&dopt=full_report&list_uids=54790) | BCL11B |  |
| 146 | C18orf25 | [ERCC6](http://www.ncbi.nlm.nih.gov/entrez/query.fcgi?db=gene&cmd=Retrieve&dopt=full_report&list_uids=2074) | ANO3 |  |
| 147 | PIK3C2G | [SENP7](http://www.ncbi.nlm.nih.gov/entrez/query.fcgi?db=gene&cmd=Retrieve&dopt=full_report&list_uids=57337) | ATP9A |  |
| 148 | NPAS3 | [TEX101](http://www.ncbi.nlm.nih.gov/entrez/query.fcgi?db=gene&cmd=Retrieve&dopt=full_report&list_uids=83639) | PTEN |  |
| 149 | FAM49B | [BAG3](http://www.ncbi.nlm.nih.gov/entrez/query.fcgi?db=gene&cmd=Retrieve&dopt=full_report&list_uids=9531) | EIF5B |  |
| 150 | KATNA1 | [LEPR](http://www.ncbi.nlm.nih.gov/entrez/query.fcgi?db=gene&cmd=Retrieve&dopt=full_report&list_uids=3953) | COL8A1 |  |
| 151 | SLFN5 | [RAP2C](http://www.ncbi.nlm.nih.gov/entrez/query.fcgi?db=gene&cmd=Retrieve&dopt=full_report&list_uids=57826) | HSPA9 |  |
| 152 | RGPD2 | [UBE2E3](http://www.ncbi.nlm.nih.gov/entrez/query.fcgi?db=gene&cmd=Retrieve&dopt=full_report&list_uids=10477) | ATMIN |  |
| 153 | PELO | [MED17](http://www.ncbi.nlm.nih.gov/entrez/query.fcgi?db=gene&cmd=Retrieve&dopt=full_report&list_uids=9440) | DLEU1 |  |
| 154 | LRRTM3 | [ZNF680](http://www.ncbi.nlm.nih.gov/entrez/query.fcgi?db=gene&cmd=Retrieve&dopt=full_report&list_uids=340252) | PICALM |  |
| 155 | PAX3 | [IPO11](http://www.ncbi.nlm.nih.gov/entrez/query.fcgi?db=gene&cmd=Retrieve&dopt=full_report&list_uids=51194) | HNRNPA2B1 |  |
| 156 | YWHAB | [COX18](http://www.ncbi.nlm.nih.gov/entrez/query.fcgi?db=gene&cmd=Retrieve&dopt=full_report&list_uids=285521) | LHX1 |  |
| 157 | SPTLC3 | [CRLS1](http://www.ncbi.nlm.nih.gov/entrez/query.fcgi?db=gene&cmd=Retrieve&dopt=full_report&list_uids=54675) | CHRDL1 |  |
| 158 | ZNF695 | [CLIC4](http://www.ncbi.nlm.nih.gov/entrez/query.fcgi?db=gene&cmd=Retrieve&dopt=full_report&list_uids=25932) | SLC19A2 |  |
| 159 | RABL3 | [STX1A](http://www.ncbi.nlm.nih.gov/entrez/query.fcgi?db=gene&cmd=Retrieve&dopt=full_report&list_uids=6804) | AFF3 |  |
| 160 | AGO3 | [RPL13](http://www.ncbi.nlm.nih.gov/entrez/query.fcgi?db=gene&cmd=Retrieve&dopt=full_report&list_uids=6137) | IARS |  |
| 161 | ANP32E | [SNRNP27](http://www.ncbi.nlm.nih.gov/entrez/query.fcgi?db=gene&cmd=Retrieve&dopt=full_report&list_uids=11017) | DLGAP2 |  |
| 162 | GPATCH2L | [MAPK8IP1](http://www.ncbi.nlm.nih.gov/entrez/query.fcgi?db=gene&cmd=Retrieve&dopt=full_report&list_uids=9479) | RAD23B |  |
| 163 | PLEKHM1 | [DHTKD1](http://www.ncbi.nlm.nih.gov/entrez/query.fcgi?db=gene&cmd=Retrieve&dopt=full_report&list_uids=55526) | HOMER1 |  |
| 164 | GHITM | [ZNF443](http://www.ncbi.nlm.nih.gov/entrez/query.fcgi?db=gene&cmd=Retrieve&dopt=full_report&list_uids=10224) | SRSF10 |  |
| 165 | SURF1 | [ZNF607](http://www.ncbi.nlm.nih.gov/entrez/query.fcgi?db=gene&cmd=Retrieve&dopt=full_report&list_uids=84775) | RAB3IP |  |
| 166 | RTF1 | [GRSF1](http://www.ncbi.nlm.nih.gov/entrez/query.fcgi?db=gene&cmd=Retrieve&dopt=full_report&list_uids=2926) | PDCD6IP |  |
| 167 | SCAMP1 | [PALM2-AKAP2](http://www.ncbi.nlm.nih.gov/entrez/query.fcgi?db=gene&cmd=Retrieve&dopt=full_report&list_uids=445815) | SENP6 |  |
| 168 | WDR75 | [C11orf87](http://www.ncbi.nlm.nih.gov/entrez/query.fcgi?db=gene&cmd=Retrieve&dopt=full_report&list_uids=399947) | CDK5R1 |  |
| 169 | DIO1 | [RICTOR](http://www.ncbi.nlm.nih.gov/entrez/query.fcgi?db=gene&cmd=Retrieve&dopt=full_report&list_uids=253260) | E2F3 |  |
| 170 | PDS5B | [ALDH16A1](http://www.ncbi.nlm.nih.gov/entrez/query.fcgi?db=gene&cmd=Retrieve&dopt=full_report&list_uids=126133) | REST |  |
| 171 | DBT | [LCOR](http://www.ncbi.nlm.nih.gov/entrez/query.fcgi?db=gene&cmd=Retrieve&dopt=full_report&list_uids=84458) | G3BP2 |  |
| 172 | SEPT11 | [ZCCHC4](http://www.ncbi.nlm.nih.gov/entrez/query.fcgi?db=gene&cmd=Retrieve&dopt=full_report&list_uids=29063) | HOXC11 |  |
| 173 | DCN | [NAA30](http://www.ncbi.nlm.nih.gov/entrez/query.fcgi?db=gene&cmd=Retrieve&dopt=full_report&list_uids=122830) | RILPL1 |  |
| 174 | STX1A | [WDR47](http://www.ncbi.nlm.nih.gov/entrez/query.fcgi?db=gene&cmd=Retrieve&dopt=full_report&list_uids=22911) | ASXL3 |  |
| 175 | DCBLD2 | [SUZ12](http://www.ncbi.nlm.nih.gov/entrez/query.fcgi?db=gene&cmd=Retrieve&dopt=full_report&list_uids=23512) | EIF3J |  |
| 176 | VAT1L | [KCTD20](http://www.ncbi.nlm.nih.gov/entrez/query.fcgi?db=gene&cmd=Retrieve&dopt=full_report&list_uids=222658) | MYEF2 |  |
| 177 | ST8SIA1 | [TMEM87A](http://www.ncbi.nlm.nih.gov/entrez/query.fcgi?db=gene&cmd=Retrieve&dopt=full_report&list_uids=25963) | KIAA0087 |  |
| 178 | LAMA4 | [IKZF5](http://www.ncbi.nlm.nih.gov/entrez/query.fcgi?db=gene&cmd=Retrieve&dopt=full_report&list_uids=64376) | LIN54 |  |
| 179 | ATCAY | [C3orf72](http://www.ncbi.nlm.nih.gov/entrez/query.fcgi?db=gene&cmd=Retrieve&dopt=full_report&list_uids=401089) | RLIM |  |
| 180 | RAB4A | [FBXO11](http://www.ncbi.nlm.nih.gov/entrez/query.fcgi?db=gene&cmd=Retrieve&dopt=full_report&list_uids=80204) | LUC7L3 |  |
| 181 | TWSG1 | [SLC16A9](http://www.ncbi.nlm.nih.gov/entrez/query.fcgi?db=gene&cmd=Retrieve&dopt=full_report&list_uids=220963) | HIPK3 |  |
| 182 | SCN7A | [DCP2](http://www.ncbi.nlm.nih.gov/entrez/query.fcgi?db=gene&cmd=Retrieve&dopt=full_report&list_uids=167227) | NDN |  |
| 183 | NCKAP1 | [SCAMP1](http://www.ncbi.nlm.nih.gov/entrez/query.fcgi?db=gene&cmd=Retrieve&dopt=full_report&list_uids=9522) | FXR1 |  |
| 184 | FOXG1 | [MOSPD1](http://www.ncbi.nlm.nih.gov/entrez/query.fcgi?db=gene&cmd=Retrieve&dopt=full_report&list_uids=56180) | QKI |  |
| 185 | AMOT | [F2R](http://www.ncbi.nlm.nih.gov/entrez/query.fcgi?db=gene&cmd=Retrieve&dopt=full_report&list_uids=2149) | HNRNPA1 |  |
| 186 | TM9SF3 | [YIPF6](http://www.ncbi.nlm.nih.gov/entrez/query.fcgi?db=gene&cmd=Retrieve&dopt=full_report&list_uids=286451) | COL21A1 |  |
| 187 | KCNA3 | [MRE11A](http://www.ncbi.nlm.nih.gov/entrez/query.fcgi?db=gene&cmd=Retrieve&dopt=full_report&list_uids=4361) | LCOR |  |
| 188 | FBXO11 | [BOD1L1](http://www.ncbi.nlm.nih.gov/entrez/query.fcgi?db=gene&cmd=Retrieve&dopt=full_report&list_uids=259282) | MED13 |  |
| 189 | SOX11 | [TTL](http://www.ncbi.nlm.nih.gov/entrez/query.fcgi?db=gene&cmd=Retrieve&dopt=full_report&list_uids=150465) | DOCK4 |  |
| 190 | ZNF85 | [ATP6V1A](http://www.ncbi.nlm.nih.gov/entrez/query.fcgi?db=gene&cmd=Retrieve&dopt=full_report&list_uids=523) | KPNA4 |  |
| 191 | BICC1 | [LUC7L3](http://www.ncbi.nlm.nih.gov/entrez/query.fcgi?db=gene&cmd=Retrieve&dopt=full_report&list_uids=51747) | AEBP2 |  |
| 192 | ATP9A | [DENND1B](http://www.ncbi.nlm.nih.gov/entrez/query.fcgi?db=gene&cmd=Retrieve&dopt=full_report&list_uids=163486) | ONECUT2 |  |
| 193 | TOM1L1 | [NAA25](http://www.ncbi.nlm.nih.gov/entrez/query.fcgi?db=gene&cmd=Retrieve&dopt=full_report&list_uids=80018) | C16orf52 |  |
| 194 | CLIC4 | [LOC400682](http://www.ncbi.nlm.nih.gov/entrez/query.fcgi?db=gene&cmd=Retrieve&dopt=full_report&list_uids=400682) | PPM1F |  |
| 195 | TEX9 | [TOR1AIP1](http://www.ncbi.nlm.nih.gov/entrez/query.fcgi?db=gene&cmd=Retrieve&dopt=full_report&list_uids=26092) | EIF4A2 |  |
| 196 | KPNA4 | [DOCK3](http://www.ncbi.nlm.nih.gov/entrez/query.fcgi?db=gene&cmd=Retrieve&dopt=full_report&list_uids=1795) | ABCC9 |  |
| 197 | SMAD2 | [DCAF10](http://www.ncbi.nlm.nih.gov/entrez/query.fcgi?db=gene&cmd=Retrieve&dopt=full_report&list_uids=79269) | DYRK1A |  |
| 198 | PLXNC1 | [KMT2D](http://www.ncbi.nlm.nih.gov/entrez/query.fcgi?db=gene&cmd=Retrieve&dopt=full_report&list_uids=8085) | TTC28 |  |
| 199 | INSIG2 | [CTSV](http://www.ncbi.nlm.nih.gov/entrez/query.fcgi?db=gene&cmd=Retrieve&dopt=full_report&list_uids=1515) | CALD1 |  |
| 200 | SLC39A10 | [NEK9](http://www.ncbi.nlm.nih.gov/entrez/query.fcgi?db=gene&cmd=Retrieve&dopt=full_report&list_uids=91754) | CXXC5 |  |
| 201 | UBL3 | [ZC3H4](http://www.ncbi.nlm.nih.gov/entrez/query.fcgi?db=gene&cmd=Retrieve&dopt=full_report&list_uids=23211) | INHBA |  |
| 202 | KCTD9 | [TDRP](http://www.ncbi.nlm.nih.gov/entrez/query.fcgi?db=gene&cmd=Retrieve&dopt=full_report&list_uids=157695) | PCMT1 |  |
| 203 | FREM2 | [TUBB6](http://www.ncbi.nlm.nih.gov/entrez/query.fcgi?db=gene&cmd=Retrieve&dopt=full_report&list_uids=84617) | G6PC |  |
| 204 | SCN9A | [TMED2](http://www.ncbi.nlm.nih.gov/entrez/query.fcgi?db=gene&cmd=Retrieve&dopt=full_report&list_uids=10959) | DNAJA1 |  |
| 205 | DNAJC10 | [APPL1](http://www.ncbi.nlm.nih.gov/entrez/query.fcgi?db=gene&cmd=Retrieve&dopt=full_report&list_uids=26060) | KIAA2026 |  |
| 206 | TTBK2 | [TMA16](http://www.ncbi.nlm.nih.gov/entrez/query.fcgi?db=gene&cmd=Retrieve&dopt=full_report&list_uids=55319) | MTDH |  |
| 207 | PQLC3 | [GTF2H2](http://www.ncbi.nlm.nih.gov/entrez/query.fcgi?db=gene&cmd=Retrieve&dopt=full_report&list_uids=2966) | CORO1C |  |
| 208 | FSD1L | [GTF2H2C](http://www.ncbi.nlm.nih.gov/entrez/query.fcgi?db=gene&cmd=Retrieve&dopt=full_report&list_uids=728340) | CNOT4 |  |
| 209 | NLGN1 | [LPHN3](http://www.ncbi.nlm.nih.gov/entrez/query.fcgi?db=gene&cmd=Retrieve&dopt=full_report&list_uids=23284) | MOSPD1 |  |
| 210 | EIF1B | [ATRN](http://www.ncbi.nlm.nih.gov/entrez/query.fcgi?db=gene&cmd=Retrieve&dopt=full_report&list_uids=8455) | CUL5 |  |
| 211 | SKA3 | [KLF3](http://www.ncbi.nlm.nih.gov/entrez/query.fcgi?db=gene&cmd=Retrieve&dopt=full_report&list_uids=51274) | PCDH8 |  |
| 212 | NDN | [TACC1](http://www.ncbi.nlm.nih.gov/entrez/query.fcgi?db=gene&cmd=Retrieve&dopt=full_report&list_uids=6867) | SLC6A1 |  |
| 213 | PSMD1 | [TIA1](http://www.ncbi.nlm.nih.gov/entrez/query.fcgi?db=gene&cmd=Retrieve&dopt=full_report&list_uids=7072) | NT5C1B-RDH14 |  |
| 214 | SOWAHC | [PQLC3](http://www.ncbi.nlm.nih.gov/entrez/query.fcgi?db=gene&cmd=Retrieve&dopt=full_report&list_uids=130814) | PSIP1 |  |
| 215 | ACER3 | [NAP1L2](http://www.ncbi.nlm.nih.gov/entrez/query.fcgi?db=gene&cmd=Retrieve&dopt=full_report&list_uids=4674) | ZNF45 |  |
| 216 | ZNF84 | [FAM98A](http://www.ncbi.nlm.nih.gov/entrez/query.fcgi?db=gene&cmd=Retrieve&dopt=full_report&list_uids=25940) | FLJ00104 |  |
| 217 | AC068987.1 | [GRIK2](http://www.ncbi.nlm.nih.gov/entrez/query.fcgi?db=gene&cmd=Retrieve&dopt=full_report&list_uids=2898) | LPPR4 |  |
| 218 | MGAT4A | [TRIL](http://www.ncbi.nlm.nih.gov/entrez/query.fcgi?db=gene&cmd=Retrieve&dopt=full_report&list_uids=9865) | CDH1 |  |
| 219 | MTF2 | [RBPJ](http://www.ncbi.nlm.nih.gov/entrez/query.fcgi?db=gene&cmd=Retrieve&dopt=full_report&list_uids=3516) | PARD3B |  |
| 220 | PTPLAD2 | [PRKD3](http://www.ncbi.nlm.nih.gov/entrez/query.fcgi?db=gene&cmd=Retrieve&dopt=full_report&list_uids=23683) | RP5-850E9.3 |  |
| 221 | EP400NL | [ZMYND11](http://www.ncbi.nlm.nih.gov/entrez/query.fcgi?db=gene&cmd=Retrieve&dopt=full_report&list_uids=10771) | USP47 |  |
| 222 | DOCK3 | [KRAS](http://www.ncbi.nlm.nih.gov/entrez/query.fcgi?db=gene&cmd=Retrieve&dopt=full_report&list_uids=3845) | TLK2 |  |
| 223 | CACHD1 | [TMEM178A](http://www.ncbi.nlm.nih.gov/entrez/query.fcgi?db=gene&cmd=Retrieve&dopt=full_report&list_uids=130733) | SH3BP4 |  |
| 224 | WDR72 | [TSHZ1](http://www.ncbi.nlm.nih.gov/entrez/query.fcgi?db=gene&cmd=Retrieve&dopt=full_report&list_uids=10194) | GDI2 |  |
| 225 | BAG3 | [MKRN1](http://www.ncbi.nlm.nih.gov/entrez/query.fcgi?db=gene&cmd=Retrieve&dopt=full_report&list_uids=23608) | CHSY1 |  |
| 226 | YOD1 | [ABCD2](http://www.ncbi.nlm.nih.gov/entrez/query.fcgi?db=gene&cmd=Retrieve&dopt=full_report&list_uids=225) | EGR1 |  |
| 227 | NUP214 | [TFCP2](http://www.ncbi.nlm.nih.gov/entrez/query.fcgi?db=gene&cmd=Retrieve&dopt=full_report&list_uids=7024) | NEUROD1 |  |
| 228 | DOK6 | [GPC5](http://www.ncbi.nlm.nih.gov/entrez/query.fcgi?db=gene&cmd=Retrieve&dopt=full_report&list_uids=2262) | TET2 |  |
| 229 | DKK1 | [ABCC4](http://www.ncbi.nlm.nih.gov/entrez/query.fcgi?db=gene&cmd=Retrieve&dopt=full_report&list_uids=10257) | LRRTM3 |  |
| 230 | TRIM64B | [WWC3](http://www.ncbi.nlm.nih.gov/entrez/query.fcgi?db=gene&cmd=Retrieve&dopt=full_report&list_uids=55841) | RNF150 |  |
| 231 | FAT3 | [DHX40](http://www.ncbi.nlm.nih.gov/entrez/query.fcgi?db=gene&cmd=Retrieve&dopt=full_report&list_uids=79665) | NT5C1B |  |
| 232 | WNT3 | [EYS](http://www.ncbi.nlm.nih.gov/entrez/query.fcgi?db=gene&cmd=Retrieve&dopt=full_report&list_uids=346007) | MTF2 |  |
| 233 | PYGO1 | [CLEC7A](http://www.ncbi.nlm.nih.gov/entrez/query.fcgi?db=gene&cmd=Retrieve&dopt=full_report&list_uids=64581) | AKAP2 |  |
| 234 | RBM46 | [TMEM135](http://www.ncbi.nlm.nih.gov/entrez/query.fcgi?db=gene&cmd=Retrieve&dopt=full_report&list_uids=65084) | FOXO3 |  |
| 235 | MAST4 | [EFNA5](http://www.ncbi.nlm.nih.gov/entrez/query.fcgi?db=gene&cmd=Retrieve&dopt=full_report&list_uids=1946) | PAX3 |  |
| 236 | DYRK1A | [LIN7C](http://www.ncbi.nlm.nih.gov/entrez/query.fcgi?db=gene&cmd=Retrieve&dopt=full_report&list_uids=55327) | SETD8 |  |
| 237 | ARID2 | [NUP214](http://www.ncbi.nlm.nih.gov/entrez/query.fcgi?db=gene&cmd=Retrieve&dopt=full_report&list_uids=8021) | FBXO21 |  |
| 238 | OSBPL1A | [CHPF](http://www.ncbi.nlm.nih.gov/entrez/query.fcgi?db=gene&cmd=Retrieve&dopt=full_report&list_uids=79586) | PANK3 |  |
| 239 | ACVR2A | [TRIM13](http://www.ncbi.nlm.nih.gov/entrez/query.fcgi?db=gene&cmd=Retrieve&dopt=full_report&list_uids=10206) | FAXC |  |
| 240 | MTERFD1 | [HSPH1](http://www.ncbi.nlm.nih.gov/entrez/query.fcgi?db=gene&cmd=Retrieve&dopt=full_report&list_uids=10808) | ST8SIA3 |  |
| 241 | TCERG1 | [TNFRSF21](http://www.ncbi.nlm.nih.gov/entrez/query.fcgi?db=gene&cmd=Retrieve&dopt=full_report&list_uids=27242) | PPM1E |  |
| 242 | MXI1 | [ATG4C](http://www.ncbi.nlm.nih.gov/entrez/query.fcgi?db=gene&cmd=Retrieve&dopt=full_report&list_uids=84938) | XPO4 |  |
| 243 | ICA1L | [TJP2](http://www.ncbi.nlm.nih.gov/entrez/query.fcgi?db=gene&cmd=Retrieve&dopt=full_report&list_uids=9414) | BTG1 |  |
| 244 | SEC23IP | [HNF4G](http://www.ncbi.nlm.nih.gov/entrez/query.fcgi?db=gene&cmd=Retrieve&dopt=full_report&list_uids=3174) | FOXG1 |  |
| 245 | MTPN | [TOMM20](http://www.ncbi.nlm.nih.gov/entrez/query.fcgi?db=gene&cmd=Retrieve&dopt=full_report&list_uids=9804) | AC068987.1 |  |
| 246 | SMAD9 | [AGFG1](http://www.ncbi.nlm.nih.gov/entrez/query.fcgi?db=gene&cmd=Retrieve&dopt=full_report&list_uids=3267) | SH3PXD2A |  |
| 247 | SAMD9 | [SLC26A11](http://www.ncbi.nlm.nih.gov/entrez/query.fcgi?db=gene&cmd=Retrieve&dopt=full_report&list_uids=284129) | POLG |  |
| 248 | TTL | [PTPN4](http://www.ncbi.nlm.nih.gov/entrez/query.fcgi?db=gene&cmd=Retrieve&dopt=full_report&list_uids=5775) | WWC3 |  |
| 249 | MICB | [SIPA1L2](http://www.ncbi.nlm.nih.gov/entrez/query.fcgi?db=gene&cmd=Retrieve&dopt=full_report&list_uids=57568) | PRPF38B |  |
| 250 | SYNCRIP | [VSNL1](http://www.ncbi.nlm.nih.gov/entrez/query.fcgi?db=gene&cmd=Retrieve&dopt=full_report&list_uids=7447) | SNAP25 |  |
| 251 | CCDC132 | [LAMP2](http://www.ncbi.nlm.nih.gov/entrez/query.fcgi?db=gene&cmd=Retrieve&dopt=full_report&list_uids=3920) | FAM60A |  |
| 252 | FAM179B | [PCMT1](http://www.ncbi.nlm.nih.gov/entrez/query.fcgi?db=gene&cmd=Retrieve&dopt=full_report&list_uids=5110) | UBXN2B |  |
| 253 | CHRDL1 | [CMTM4](http://www.ncbi.nlm.nih.gov/entrez/query.fcgi?db=gene&cmd=Retrieve&dopt=full_report&list_uids=146223) | ZC3H4 |  |
| 254 | NSUN3 | [MEF2C](http://www.ncbi.nlm.nih.gov/entrez/query.fcgi?db=gene&cmd=Retrieve&dopt=full_report&list_uids=4208) | PSMF1 |  |
| 255 | ANKIB1 | [ASAP2](http://www.ncbi.nlm.nih.gov/entrez/query.fcgi?db=gene&cmd=Retrieve&dopt=full_report&list_uids=8853) | HNRNPUL1 |  |
| 256 | C5orf15 | [IKZF3](http://www.ncbi.nlm.nih.gov/entrez/query.fcgi?db=gene&cmd=Retrieve&dopt=full_report&list_uids=22806) | ANKRD50 |  |
| 257 | PALM2-AKAP2 | [SEMA6D](http://www.ncbi.nlm.nih.gov/entrez/query.fcgi?db=gene&cmd=Retrieve&dopt=full_report&list_uids=80031) | SATB1 |  |
| 258 | FBXL5 | [ZNF708](http://www.ncbi.nlm.nih.gov/entrez/query.fcgi?db=gene&cmd=Retrieve&dopt=full_report&list_uids=7562) | PLEKHM1 |  |
| 259 | ZNF302 | [ANTXR1](http://www.ncbi.nlm.nih.gov/entrez/query.fcgi?db=gene&cmd=Retrieve&dopt=full_report&list_uids=84168) | CREBZF |  |
| 260 | LPAR1 | [CADPS2](http://www.ncbi.nlm.nih.gov/entrez/query.fcgi?db=gene&cmd=Retrieve&dopt=full_report&list_uids=93664) | FN1 |  |
| 261 | WDFY4 | [SYCP2](http://www.ncbi.nlm.nih.gov/entrez/query.fcgi?db=gene&cmd=Retrieve&dopt=full_report&list_uids=10388) | NCOA7 |  |
| 262 | BTC | [C1orf27](http://www.ncbi.nlm.nih.gov/entrez/query.fcgi?db=gene&cmd=Retrieve&dopt=full_report&list_uids=54953) | RAPGEF6 |  |
| 263 | GUCY1A3 | [ZNF92](http://www.ncbi.nlm.nih.gov/entrez/query.fcgi?db=gene&cmd=Retrieve&dopt=full_report&list_uids=168374) | ATCAY |  |
| 264 | TBRG1 | [TBC1D30](http://www.ncbi.nlm.nih.gov/entrez/query.fcgi?db=gene&cmd=Retrieve&dopt=full_report&list_uids=23329) | SLITRK3 |  |
| 265 | SLC7A11 | [PDS5B](http://www.ncbi.nlm.nih.gov/entrez/query.fcgi?db=gene&cmd=Retrieve&dopt=full_report&list_uids=23047) | SLC39A10 |  |
| 266 | PIKFYVE | [PDCD6IP](http://www.ncbi.nlm.nih.gov/entrez/query.fcgi?db=gene&cmd=Retrieve&dopt=full_report&list_uids=10015) | CTC-432M15.3 |  |
| 267 | PROSER1 | [REST](http://www.ncbi.nlm.nih.gov/entrez/query.fcgi?db=gene&cmd=Retrieve&dopt=full_report&list_uids=5978) | PURB |  |
| 268 | SIGLEC8 | [ELK4](http://www.ncbi.nlm.nih.gov/entrez/query.fcgi?db=gene&cmd=Retrieve&dopt=full_report&list_uids=2005) | YY1 |  |
| 269 | PNRC2 | [UNC119B](http://www.ncbi.nlm.nih.gov/entrez/query.fcgi?db=gene&cmd=Retrieve&dopt=full_report&list_uids=84747) | ZFP69B |  |
| 270 | AEBP1 | [SLC35G3](http://www.ncbi.nlm.nih.gov/entrez/query.fcgi?db=gene&cmd=Retrieve&dopt=full_report&list_uids=146861) | CLOCK |  |
| 271 | AGGF1 | [LBR](http://www.ncbi.nlm.nih.gov/entrez/query.fcgi?db=gene&cmd=Retrieve&dopt=full_report&list_uids=3930) | RPGRIP1L |  |
| 272 | KLF12 | [PDE5A](http://www.ncbi.nlm.nih.gov/entrez/query.fcgi?db=gene&cmd=Retrieve&dopt=full_report&list_uids=8654) | STRN |  |
| 273 | ZNF615 | [OTUD7B](http://www.ncbi.nlm.nih.gov/entrez/query.fcgi?db=gene&cmd=Retrieve&dopt=full_report&list_uids=56957) | TCF7L2 |  |
| 274 | PURB | [MAF](http://www.ncbi.nlm.nih.gov/entrez/query.fcgi?db=gene&cmd=Retrieve&dopt=full_report&list_uids=4094) | YWHAH |  |
| 275 | RILPL1 | [ITGAV](http://www.ncbi.nlm.nih.gov/entrez/query.fcgi?db=gene&cmd=Retrieve&dopt=full_report&list_uids=3685) | ZIC1 |  |
| 276 | ESCO1 | [TMEM167A](http://www.ncbi.nlm.nih.gov/entrez/query.fcgi?db=gene&cmd=Retrieve&dopt=full_report&list_uids=153339) | WASF3 |  |
| 277 | AEBP2 | [C18orf63](http://www.ncbi.nlm.nih.gov/entrez/query.fcgi?db=gene&cmd=Retrieve&dopt=full_report&list_uids=644041) | GOLGA1 |  |
| 278 | RBM20 | [CHRDL1](http://www.ncbi.nlm.nih.gov/entrez/query.fcgi?db=gene&cmd=Retrieve&dopt=full_report&list_uids=91851) | ROBO1 |  |
| 279 | PGM2L1 | [ZNF257](http://www.ncbi.nlm.nih.gov/entrez/query.fcgi?db=gene&cmd=Retrieve&dopt=full_report&list_uids=113835) | RCAN1 |  |
| 280 | ZBTB8B | [WHSC1](http://www.ncbi.nlm.nih.gov/entrez/query.fcgi?db=gene&cmd=Retrieve&dopt=full_report&list_uids=7468) | RAP1B |  |
| 281 | NOVA1 | [CCDC67](http://www.ncbi.nlm.nih.gov/entrez/query.fcgi?db=gene&cmd=Retrieve&dopt=full_report&list_uids=159989) | EPHA5 |  |
| 282 | SMC2 | [TLK1](http://www.ncbi.nlm.nih.gov/entrez/query.fcgi?db=gene&cmd=Retrieve&dopt=full_report&list_uids=9874) | TMEM68 |  |
| 283 | PDCD6IP | [NALCN](http://www.ncbi.nlm.nih.gov/entrez/query.fcgi?db=gene&cmd=Retrieve&dopt=full_report&list_uids=259232) | CSGALNACT2 |  |
| 284 | ARNTL2 | [DDX53](http://www.ncbi.nlm.nih.gov/entrez/query.fcgi?db=gene&cmd=Retrieve&dopt=full_report&list_uids=168400) | MTF1 |  |
| 285 | MAN1A1 | [DNA2](http://www.ncbi.nlm.nih.gov/entrez/query.fcgi?db=gene&cmd=Retrieve&dopt=full_report&list_uids=1763) | FSD1L |  |
| 286 | MARVELD2 | [KIF3C](http://www.ncbi.nlm.nih.gov/entrez/query.fcgi?db=gene&cmd=Retrieve&dopt=full_report&list_uids=3797) | RALGPS1 |  |
| 287 | C8orf86 | [PTPRN2](http://www.ncbi.nlm.nih.gov/entrez/query.fcgi?db=gene&cmd=Retrieve&dopt=full_report&list_uids=5799) | SEPT11 |  |
| 288 | ZNF207 | [B3GALTL](http://www.ncbi.nlm.nih.gov/entrez/query.fcgi?db=gene&cmd=Retrieve&dopt=full_report&list_uids=145173) | UBE2E3 |  |
| 289 | FIBP | [GPR126](http://www.ncbi.nlm.nih.gov/entrez/query.fcgi?db=gene&cmd=Retrieve&dopt=full_report&list_uids=57211) | USP7 |  |
| 290 | TP53TG3C | [PCGF3](http://www.ncbi.nlm.nih.gov/entrez/query.fcgi?db=gene&cmd=Retrieve&dopt=full_report&list_uids=10336) | SHISA9 |  |
| 291 | TP53TG3 | [ZNF273](http://www.ncbi.nlm.nih.gov/entrez/query.fcgi?db=gene&cmd=Retrieve&dopt=full_report&list_uids=10793) | GBX2 |  |
| 292 | PDE5A | [SLC4A5](http://www.ncbi.nlm.nih.gov/entrez/query.fcgi?db=gene&cmd=Retrieve&dopt=full_report&list_uids=57835) | EPHA7 |  |
| 293 | TIFA | [USP37](http://www.ncbi.nlm.nih.gov/entrez/query.fcgi?db=gene&cmd=Retrieve&dopt=full_report&list_uids=57695) | NLGN1 |  |
| 294 | ZFHX2 | [HMGB2](http://www.ncbi.nlm.nih.gov/entrez/query.fcgi?db=gene&cmd=Retrieve&dopt=full_report&list_uids=3148) | HIF1A |  |
| 295 | TMEM170B | [ASXL3](http://www.ncbi.nlm.nih.gov/entrez/query.fcgi?db=gene&cmd=Retrieve&dopt=full_report&list_uids=80816) | FOXO4 |  |
| 296 | ZNF141 | [DNAJC5](http://www.ncbi.nlm.nih.gov/entrez/query.fcgi?db=gene&cmd=Retrieve&dopt=full_report&list_uids=80331) | ABCC1 |  |
| 297 | GPR126 | [ZNF100](http://www.ncbi.nlm.nih.gov/entrez/query.fcgi?db=gene&cmd=Retrieve&dopt=full_report&list_uids=163227) | GRIK2 |  |
| 298 | DYNC1LI2 | [43350](http://www.ncbi.nlm.nih.gov/entrez/query.fcgi?db=gene&cmd=Retrieve&dopt=full_report&list_uids=989) | SEPT6 |  |
| 299 | BRMS1L | [C8orf86](http://www.ncbi.nlm.nih.gov/entrez/query.fcgi?db=gene&cmd=Retrieve&dopt=full_report&list_uids=389649) | KCNB1 |  |
| 300 | MGA | [ZNF431](http://www.ncbi.nlm.nih.gov/entrez/query.fcgi?db=gene&cmd=Retrieve&dopt=full_report&list_uids=170959) | AP3M1 |  |
| 301 | TP53TG3D | [ELOVL7](http://www.ncbi.nlm.nih.gov/entrez/query.fcgi?db=gene&cmd=Retrieve&dopt=full_report&list_uids=79993) | KLF12 |  |
| 302 | ZNF211 | [TOPORS](http://www.ncbi.nlm.nih.gov/entrez/query.fcgi?db=gene&cmd=Retrieve&dopt=full_report&list_uids=10210) | ROCK1 |  |
| 303 | RIPK2 | [LMO7](http://www.ncbi.nlm.nih.gov/entrez/query.fcgi?db=gene&cmd=Retrieve&dopt=full_report&list_uids=4008) | ELMOD1 |  |
| 304 | LBR | [ST18](http://www.ncbi.nlm.nih.gov/entrez/query.fcgi?db=gene&cmd=Retrieve&dopt=full_report&list_uids=9705) | MACF1 |  |
| 305 | USP24 | [C6orf25](http://www.ncbi.nlm.nih.gov/entrez/query.fcgi?db=gene&cmd=Retrieve&dopt=full_report&list_uids=80739) | REEP5 |  |
| 306 | EIF3J | [GNL1](http://www.ncbi.nlm.nih.gov/entrez/query.fcgi?db=gene&cmd=Retrieve&dopt=full_report&list_uids=2794) | CELF2 |  |
| 307 | ZNF681 | [TMEM170B](http://www.ncbi.nlm.nih.gov/entrez/query.fcgi?db=gene&cmd=Retrieve&dopt=full_report&list_uids=100113407) | FREM2 |  |
| 308 | C14orf37 | [TM4SF18](http://www.ncbi.nlm.nih.gov/entrez/query.fcgi?db=gene&cmd=Retrieve&dopt=full_report&list_uids=116441) | PPP1CB |  |
| 309 | SLC35B3 | [CDK5R1](http://www.ncbi.nlm.nih.gov/entrez/query.fcgi?db=gene&cmd=Retrieve&dopt=full_report&list_uids=8851) | NCAM1 |  |
| 310 | ZNF268 | [PPM1E](http://www.ncbi.nlm.nih.gov/entrez/query.fcgi?db=gene&cmd=Retrieve&dopt=full_report&list_uids=22843) | FTO |  |
| 311 | DACT1 | [CTXN3](http://www.ncbi.nlm.nih.gov/entrez/query.fcgi?db=gene&cmd=Retrieve&dopt=full_report&list_uids=613212) | KCNH5 |  |
| 312 | ZNF605 | [TXNDC17](http://www.ncbi.nlm.nih.gov/entrez/query.fcgi?db=gene&cmd=Retrieve&dopt=full_report&list_uids=84817) | ELAVL2 |  |
| 313 | ASXL1 | [IKBIP](http://www.ncbi.nlm.nih.gov/entrez/query.fcgi?db=gene&cmd=Retrieve&dopt=full_report&list_uids=121457) | NIPBL |  |
| 314 | IPO8 | [MBD2](http://www.ncbi.nlm.nih.gov/entrez/query.fcgi?db=gene&cmd=Retrieve&dopt=full_report&list_uids=8932) | MID2 |  |
| 315 | MMP16 | [GKAP1](http://www.ncbi.nlm.nih.gov/entrez/query.fcgi?db=gene&cmd=Retrieve&dopt=full_report&list_uids=80318) | EEA1 |  |
| 316 | GMFB | [CSGALNACT2](http://www.ncbi.nlm.nih.gov/entrez/query.fcgi?db=gene&cmd=Retrieve&dopt=full_report&list_uids=55454) | TRERF1 |  |
| 317 | TMOD4 | [HPS3](http://www.ncbi.nlm.nih.gov/entrez/query.fcgi?db=gene&cmd=Retrieve&dopt=full_report&list_uids=84343) | ATP1B1 |  |
| 318 | C12orf4 | [FSD1L](http://www.ncbi.nlm.nih.gov/entrez/query.fcgi?db=gene&cmd=Retrieve&dopt=full_report&list_uids=83856) | NOVA1 |  |
| 319 | PLA2G2C | [UBA6](http://www.ncbi.nlm.nih.gov/entrez/query.fcgi?db=gene&cmd=Retrieve&dopt=full_report&list_uids=55236) | USP38 |  |
| 320 | AKAP10 | [DHX33](http://www.ncbi.nlm.nih.gov/entrez/query.fcgi?db=gene&cmd=Retrieve&dopt=full_report&list_uids=56919) | SSH1 |  |
| 321 | RAD50 | [NOM1](http://www.ncbi.nlm.nih.gov/entrez/query.fcgi?db=gene&cmd=Retrieve&dopt=full_report&list_uids=64434) | DENR |  |
| 322 | ATAD2 | [TMED10](http://www.ncbi.nlm.nih.gov/entrez/query.fcgi?db=gene&cmd=Retrieve&dopt=full_report&list_uids=10972) | SNX27 |  |
| 323 | SENP6 | [TXNDC16](http://www.ncbi.nlm.nih.gov/entrez/query.fcgi?db=gene&cmd=Retrieve&dopt=full_report&list_uids=57544) | RBM38 |  |
| 324 | ATPAF1 | [KATNA1](http://www.ncbi.nlm.nih.gov/entrez/query.fcgi?db=gene&cmd=Retrieve&dopt=full_report&list_uids=11104) | REEP1 |  |
| 325 | NALCN | [RAB17](http://www.ncbi.nlm.nih.gov/entrez/query.fcgi?db=gene&cmd=Retrieve&dopt=full_report&list_uids=64284) | GOLIM4 |  |
| 326 | KRAS | [EIF4A2](http://www.ncbi.nlm.nih.gov/entrez/query.fcgi?db=gene&cmd=Retrieve&dopt=full_report&list_uids=1974) | NAA30 |  |
| 327 | SFT2D3 | [DLX3](http://www.ncbi.nlm.nih.gov/entrez/query.fcgi?db=gene&cmd=Retrieve&dopt=full_report&list_uids=1747) | DESI2 |  |
| 328 | E2F3 | [SFMBT2](http://www.ncbi.nlm.nih.gov/entrez/query.fcgi?db=gene&cmd=Retrieve&dopt=full_report&list_uids=57713) | KCNA3 |  |
| 329 | ZSWIM6 | [ZNF714](http://www.ncbi.nlm.nih.gov/entrez/query.fcgi?db=gene&cmd=Retrieve&dopt=full_report&list_uids=148206) | TCF12 |  |
| 330 | NFIB | [STAU2](http://www.ncbi.nlm.nih.gov/entrez/query.fcgi?db=gene&cmd=Retrieve&dopt=full_report&list_uids=27067) | SLC4A4 |  |
| 331 | ITM2B | [BDKRB2](http://www.ncbi.nlm.nih.gov/entrez/query.fcgi?db=gene&cmd=Retrieve&dopt=full_report&list_uids=624) | ESRRG |  |
| 332 | ZNF256 | [CNTN3](http://www.ncbi.nlm.nih.gov/entrez/query.fcgi?db=gene&cmd=Retrieve&dopt=full_report&list_uids=5067) | C2orf71 |  |
| 333 | LYRM9 | [ATG7](http://www.ncbi.nlm.nih.gov/entrez/query.fcgi?db=gene&cmd=Retrieve&dopt=full_report&list_uids=10533) | ZDHHC3 |  |
| 334 | WDR19 | [HERPUD1](http://www.ncbi.nlm.nih.gov/entrez/query.fcgi?db=gene&cmd=Retrieve&dopt=full_report&list_uids=9709) | DGKH |  |
| 335 | TMEM65 | [ZNF695](http://www.ncbi.nlm.nih.gov/entrez/query.fcgi?db=gene&cmd=Retrieve&dopt=full_report&list_uids=57116) | KIF2A |  |
| 336 | GOPC | [SDR42E1](http://www.ncbi.nlm.nih.gov/entrez/query.fcgi?db=gene&cmd=Retrieve&dopt=full_report&list_uids=93517) | ZFAND5 |  |
| 337 | EIF5B | [MS4A2](http://www.ncbi.nlm.nih.gov/entrez/query.fcgi?db=gene&cmd=Retrieve&dopt=full_report&list_uids=2206) | MGAT4A |  |
| 338 | ANKRD50 | [SOS1](http://www.ncbi.nlm.nih.gov/entrez/query.fcgi?db=gene&cmd=Retrieve&dopt=full_report&list_uids=6654) | DST |  |
| 339 | SLC33A1 | [TMEM65](http://www.ncbi.nlm.nih.gov/entrez/query.fcgi?db=gene&cmd=Retrieve&dopt=full_report&list_uids=157378) | PDPN |  |
| 340 | TOMM70A | [TCF7L2](http://www.ncbi.nlm.nih.gov/entrez/query.fcgi?db=gene&cmd=Retrieve&dopt=full_report&list_uids=6934) | RBM20 |  |
| 341 | WDR77 | [MFSD6](http://www.ncbi.nlm.nih.gov/entrez/query.fcgi?db=gene&cmd=Retrieve&dopt=full_report&list_uids=54842) | APOOL |  |
| 342 | ZNF257 | [BRMS1L](http://www.ncbi.nlm.nih.gov/entrez/query.fcgi?db=gene&cmd=Retrieve&dopt=full_report&list_uids=84312) | GALNTL6 |  |
| 343 | WNK3 | [NCK1](http://www.ncbi.nlm.nih.gov/entrez/query.fcgi?db=gene&cmd=Retrieve&dopt=full_report&list_uids=4690) | LONRF3 |  |
| 344 | SENP5 | [C8orf34](http://www.ncbi.nlm.nih.gov/entrez/query.fcgi?db=gene&cmd=Retrieve&dopt=full_report&list_uids=116328) | AMOT |  |
| 345 | INTS8 | [LRP6](http://www.ncbi.nlm.nih.gov/entrez/query.fcgi?db=gene&cmd=Retrieve&dopt=full_report&list_uids=4040) | SLC30A5 |  |
| 346 | RIMS2 | [LMBR1](http://www.ncbi.nlm.nih.gov/entrez/query.fcgi?db=gene&cmd=Retrieve&dopt=full_report&list_uids=64327) | TRMT1L |  |
| 347 | RPAIN | [NIPBL](http://www.ncbi.nlm.nih.gov/entrez/query.fcgi?db=gene&cmd=Retrieve&dopt=full_report&list_uids=25836) | STOX2 |  |
| 348 | ANO3 | [PYGO1](http://www.ncbi.nlm.nih.gov/entrez/query.fcgi?db=gene&cmd=Retrieve&dopt=full_report&list_uids=26108) | PIKFYVE |  |
| 349 | SLC4A4 | [CD36](http://www.ncbi.nlm.nih.gov/entrez/query.fcgi?db=gene&cmd=Retrieve&dopt=full_report&list_uids=948) | IKZF3 |  |
| 350 | AKAP2 | [FNDC1](http://www.ncbi.nlm.nih.gov/entrez/query.fcgi?db=gene&cmd=Retrieve&dopt=full_report&list_uids=84624) | ATP6V1A |  |
| 351 | SLC4A5 | [AGO4](http://www.ncbi.nlm.nih.gov/entrez/query.fcgi?db=gene&cmd=Retrieve&dopt=full_report&list_uids=192670) | ANK3 |  |
| 352 | CROT | [SKAP2](http://www.ncbi.nlm.nih.gov/entrez/query.fcgi?db=gene&cmd=Retrieve&dopt=full_report&list_uids=8935) | ZBTB16 |  |
| 353 | ZNF43 | [KIAA0319](http://www.ncbi.nlm.nih.gov/entrez/query.fcgi?db=gene&cmd=Retrieve&dopt=full_report&list_uids=9856) | RUNX1 |  |
| 354 | SIK2 | [CENPP](http://www.ncbi.nlm.nih.gov/entrez/query.fcgi?db=gene&cmd=Retrieve&dopt=full_report&list_uids=401541) | ZSWIM6 |  |
| 355 | SUB1 | [RLIM](http://www.ncbi.nlm.nih.gov/entrez/query.fcgi?db=gene&cmd=Retrieve&dopt=full_report&list_uids=51132) | TARBP2 |  |
| 356 | FLVCR1 | [ITPA](http://www.ncbi.nlm.nih.gov/entrez/query.fcgi?db=gene&cmd=Retrieve&dopt=full_report&list_uids=3704) | STRBP |  |
| 357 | DNAJC7 | [ACKR3](http://www.ncbi.nlm.nih.gov/entrez/query.fcgi?db=gene&cmd=Retrieve&dopt=full_report&list_uids=57007) | PCNXL2 |  |
| 358 | FAM102B | [ID4](http://www.ncbi.nlm.nih.gov/entrez/query.fcgi?db=gene&cmd=Retrieve&dopt=full_report&list_uids=3400) | ABL2 |  |
| 359 | TNFSF11 | [SLC39A10](http://www.ncbi.nlm.nih.gov/entrez/query.fcgi?db=gene&cmd=Retrieve&dopt=full_report&list_uids=57181) | ELL |  |
| 360 | CNKSR3 | [LOC101060596](http://www.ncbi.nlm.nih.gov/entrez/query.fcgi?db=gene&cmd=Retrieve&dopt=full_report&list_uids=101060596) | EIF4E3 |  |
| 361 | PRKRA | [CHRAC1](http://www.ncbi.nlm.nih.gov/entrez/query.fcgi?db=gene&cmd=Retrieve&dopt=full_report&list_uids=54108) | SDC2 |  |
| 362 | DISC1 | [NF1](http://www.ncbi.nlm.nih.gov/entrez/query.fcgi?db=gene&cmd=Retrieve&dopt=full_report&list_uids=4763) | PPARGC1A |  |
| 363 | G3BP2 | [BICD2](http://www.ncbi.nlm.nih.gov/entrez/query.fcgi?db=gene&cmd=Retrieve&dopt=full_report&list_uids=23299) | HIST1H2BF |  |
| 364 | BTN3A1 | [OTUD1](http://www.ncbi.nlm.nih.gov/entrez/query.fcgi?db=gene&cmd=Retrieve&dopt=full_report&list_uids=220213) | SCUBE3 |  |
| 365 | MED17 | [RTN1](http://www.ncbi.nlm.nih.gov/entrez/query.fcgi?db=gene&cmd=Retrieve&dopt=full_report&list_uids=6252) | EPC1 |  |
| 366 | C4orf22 | [COL4A5](http://www.ncbi.nlm.nih.gov/entrez/query.fcgi?db=gene&cmd=Retrieve&dopt=full_report&list_uids=1287) | TGFBR2 |  |
| 367 | NEXN | [SLC6A1](http://www.ncbi.nlm.nih.gov/entrez/query.fcgi?db=gene&cmd=Retrieve&dopt=full_report&list_uids=6529) | TMEM167A |  |
| 368 | G6PC | [CERS6](http://www.ncbi.nlm.nih.gov/entrez/query.fcgi?db=gene&cmd=Retrieve&dopt=full_report&list_uids=253782) | CDS2 |  |
| 369 | SEC31B | [CALHM2](http://www.ncbi.nlm.nih.gov/entrez/query.fcgi?db=gene&cmd=Retrieve&dopt=full_report&list_uids=51063) | G3BP1 |  |
| 370 | EMC9 | [ISLR2](http://www.ncbi.nlm.nih.gov/entrez/query.fcgi?db=gene&cmd=Retrieve&dopt=full_report&list_uids=57611) | RBM39 |  |
| 371 | DAB1 | [SLC19A2](http://www.ncbi.nlm.nih.gov/entrez/query.fcgi?db=gene&cmd=Retrieve&dopt=full_report&list_uids=10560) | HIVEP3 |  |
| 372 | CERS6 | [MXI1](http://www.ncbi.nlm.nih.gov/entrez/query.fcgi?db=gene&cmd=Retrieve&dopt=full_report&list_uids=4601) | GAB1 |  |
| 373 | CLK4 | [PDHA1](http://www.ncbi.nlm.nih.gov/entrez/query.fcgi?db=gene&cmd=Retrieve&dopt=full_report&list_uids=5160) | SOX6 |  |
| 374 | C11orf87 | [RBM39](http://www.ncbi.nlm.nih.gov/entrez/query.fcgi?db=gene&cmd=Retrieve&dopt=full_report&list_uids=9584) | SCN2A |  |
| 375 | USP3 | [AAK1](http://www.ncbi.nlm.nih.gov/entrez/query.fcgi?db=gene&cmd=Retrieve&dopt=full_report&list_uids=22848) | MEIS1 |  |
| 376 | UNC13C | [HMOX1](http://www.ncbi.nlm.nih.gov/entrez/query.fcgi?db=gene&cmd=Retrieve&dopt=full_report&list_uids=3162) | CSMD2 |  |
| 377 | GTF2H2 | [DST](http://www.ncbi.nlm.nih.gov/entrez/query.fcgi?db=gene&cmd=Retrieve&dopt=full_report&list_uids=667) | VPS29 |  |
| 378 | CELF2 | [TMEM33](http://www.ncbi.nlm.nih.gov/entrez/query.fcgi?db=gene&cmd=Retrieve&dopt=full_report&list_uids=55161) | ZNF516 |  |
| 379 | TMA16 | [STAG2](http://www.ncbi.nlm.nih.gov/entrez/query.fcgi?db=gene&cmd=Retrieve&dopt=full_report&list_uids=10735) | SLC23A2 |  |
| 380 | TREM1 | [COL6A6](http://www.ncbi.nlm.nih.gov/entrez/query.fcgi?db=gene&cmd=Retrieve&dopt=full_report&list_uids=131873) | CAND1 |  |
| 381 | FAR2 |  | TAF1D |  |
| 382 | ZNF493 |  | MED1 |  |
| 383 | PTEN |  | SUZ12 |  |
| 384 | RP1-27O5.3 |  | CADPS2 |  |
| 385 | LCA5 |  | ZNF280C |  |
| 386 | CPSF2 |  | ZNRF2 |  |
| 387 | DCLK3 |  | GATAD2B |  |
| 388 | DCP2 |  | CAMK1D |  |
| 389 | KCNH5 |  | PEAK1 |  |
| 390 | MTTP |  | RIMKLA |  |
| 391 | DLG2 |  | C18orf25 |  |
| 392 | AC008060.7 |  | MON2 |  |
| 393 | WWC3 |  | LRRC40 |  |
| 394 | OPA3 |  | TMEM246 |  |
| 395 | PRPF38B |  | PRR14L |  |
| 396 | REST |  | MFSD6 |  |
| 397 | LUC7L3 |  | AMD1 |  |
| 398 | PPARGC1A |  | KCTD16 |  |
| 399 | SOX6 |  | ARHGAP32 |  |
| 400 | CALD1 |  | EHD4 |  |
| 401 | UFSP2 |  | PHF1 |  |
| 402 | ZNF562 |  | MRE11A |  |
| 403 | RBM33 |  | RANBP2 |  |
| 404 | TRIP12 |  | TIAL1 |  |
| 405 | ELF5 |  | ASAP2 |  |
| 406 | FZD4 |  | USP37 |  |
| 407 | SPECC1 |  | THRA |  |
| 408 | TDRP |  | TNIK |  |
| 409 | THBS1 |  | AZIN1 |  |
| 410 | GATSL2 |  | ZNF423 |  |
| 411 | SNX27 |  | EML6 |  |
| 412 | GLS2 |  | ZBTB20 |  |
| 413 | NT5DC1 |  | PCDH17 |  |
| 414 | APRT |  | WDFY4 |  |
| 415 | CORO1C |  | POU3F3 |  |
| 416 | KIAA1549 |  | AGO2 |  |
| 417 | C10orf118 |  | JMJD1C |  |
| 418 | PLD1 |  | SOX11 |  |
| 419 | ZNF385D |  | SUV420H1 |  |
| 420 | C4orf46 |  | PCDH1 |  |
| 421 | UBA3 |  | ZNF704 |  |
| 422 | FEM1C |  | ATAD2B |  |
| 423 | TMSB4X |  | FAM73B |  |
| 424 | PDSS2 |  | FOXP1 |  |
| 425 | TNRC6A |  | SIK2 |  |
| 426 | USP9Y |  | FNDC3B |  |
| 427 | DNM3 |  | ATRN |  |
| 428 | GATAD2B |  | ARHGAP12 |  |
| 429 | ZDHHC21 |  | ZNF445 |  |
| 430 | MFSD6 |  | KMT2D |  |
| 431 | ABL2 |  | TACC1 |  |
| 432 | TDO2 |  | FAF2 |  |
| 433 | ATF1 |  | ADCYAP1 |  |
| 434 | GAS2 |  | DYNC1LI2 |  |
| 435 | EDIL3 |  | ASF1A |  |
| 436 | NUDT3 |  | PMPCB |  |
| 437 | TEX14 |  | MYPN |  |
| 438 | ADAM12 |  | ZFHX2 |  |
| 439 | IAPP |  | RASD1 |  |
| 440 | BRWD1 |  | CNNM2 |  |
| 441 | MAP1B |  | SUPT3H |  |
| 442 | DCTN2 |  | ACVR2A |  |
| 443 | ANKS3 |  | NUFIP2 |  |
| 444 | PLXNB1 |  | METTL8 |  |
| 445 | THUMPD1 |  | MSH6 |  |
| 446 | HNRNPA2B1 |  | ARID1A |  |
| 447 | OPCML |  | YWHAB |  |
| 448 | SLC35G3 |  | PDS5B |  |
| 449 | ADAMTS6 |  | ATG7 |  |
| 450 | DPY19L4 |  | PTCD1 |  |
| 451 | HERC4 |  | FAM149B1 |  |
| 452 | DOCK5 |  | HARS |  |
| 453 | RYR1 |  | AHSA2 |  |
| 454 | TSG101 |  | SLC35C2 |  |
| 455 | PKP1 |  | CMC1 |  |
| 456 | SYT15 |  | BCL9L |  |
| 457 | AKAP11 |  | SHMT1 |  |
| 458 | ZNF26 |  | CNTNAP5 |  |
| 459 | RIMKLA |  | CLU |  |
| 460 | LRRD1 |  | DUSP3 |  |
| 461 | RHOXF2B |  | ATP5J2-PTCD1 |  |
| 462 | DCLK1 |  |  |  |
| 463 | CDC5L |  |  |  |
| 464 | RHOT1 |  |  |  |
| 465 | VPS13C |  |  |  |
| 466 | BAI3 |  |  |  |
| 467 | CUX1 |  |  |  |
| 468 | EXTL2 |  |  |  |
| 469 | TMEM26 |  |  |  |
| 470 | AMOTL1 |  |  |  |
| 471 | ESRP1 |  |  |  |
| 472 | ST8SIA3 |  |  |  |
| 473 | BCL11B |  |  |  |
| 474 | CBFB |  |  |  |
| 475 | FGF20 |  |  |  |
| 476 | HNRNPA3 |  |  |  |
| 477 | SCFD1 |  |  |  |
| 478 | AFF3 |  |  |  |
| 479 | ZNF486 |  |  |  |
| 480 | FBXO48 |  |  |  |
| 481 | KIF3C |  |  |  |
| 482 | PICALM |  |  |  |
| 483 | NUDT15 |  |  |  |
| 484 | HYOU1 |  |  |  |
| 485 | FAM49A |  |  |  |
| 486 | KLF5 |  |  |  |
| 487 | SPSB4 |  |  |  |
| 488 | ASB9 |  |  |  |
| 489 | ACVR2B |  |  |  |
| 490 | SEPT7 |  |  |  |
| 491 | LHPP |  |  |  |
| 492 | CANX |  |  |  |
| 493 | HIF1AN |  |  |  |
| 494 | ZPBP2 |  |  |  |
| 495 | NPTN |  |  |  |
| 496 | METTL8 |  |  |  |
| 497 | TMEM64 |  |  |  |
| 498 | AKAP9 |  |  |  |
| 499 | ZNF410 |  |  |  |
| 500 | KCTD16 |  |  |  |
| 501 | B3GALNT1 |  |  |  |
| 502 | MBD2 |  |  |  |
| 503 | CXXC5 |  |  |  |
| 504 | ZIC1 |  |  |  |
| 505 | USP45 |  |  |  |
| 506 | SNX33 |  |  |  |
| 507 | DYNLT3 |  |  |  |
| 508 | NSUN2 |  |  |  |
| 509 | HHIP |  |  |  |
| 510 | SGK1 |  |  |  |
| 511 | RBM38 |  |  |  |
| 512 | MPP6 |  |  |  |
| 513 | TBC1D15 |  |  |  |
| 514 | SMCHD1 |  |  |  |
| 515 | THOC1 |  |  |  |
| 516 | PTPRD |  |  |  |
| 517 | NDUFA9 |  |  |  |
| 518 | BMPER |  |  |  |
| 519 | PANK3 |  |  |  |
| 520 | SUV420H1 |  |  |  |
| 521 | ARMC10 |  |  |  |
| 522 | MTURN |  |  |  |
| 523 | PRR14L |  |  |  |
| 524 | CMC1 |  |  |  |
| 525 | RAPGEF6 |  |  |  |
| 526 | SCYL3 |  |  |  |
| 527 | SNRNP27 |  |  |  |
| 528 | TTC39B |  |  |  |
| 529 | TXNDC16 |  |  |  |
| 530 | FAM168B |  |  |  |
| 531 | CRLS1 |  |  |  |
| 532 | CTNND2 |  |  |  |
| 533 | FOXP1 |  |  |  |
| 534 | AP2B1 |  |  |  |
| 535 | SLC30A1 |  |  |  |
| 536 | WDR82 |  |  |  |
| 537 | LSM14A |  |  |  |
| 538 | CXorf24 |  |  |  |
| 539 | CXCL14 |  |  |  |
| 540 | PCGF5 |  |  |  |
| 541 | GTF2IRD2B |  |  |  |
| 542 | PEX13 |  |  |  |
| 543 | BTBD11 |  |  |  |
| 544 | ST8SIA6 |  |  |  |
| 545 | CAV3 |  |  |  |
| 546 | AGO4 |  |  |  |
| 547 | STAG2 |  |  |  |
| 548 | ZMAT3 |  |  |  |
| 549 | STAM2 |  |  |  |
| 550 | EMC3 |  |  |  |
| 551 | RAB11FIP5 |  |  |  |
| 552 | MTRR |  |  |  |
| 553 | AMD1 |  |  |  |
| 554 | TAOK1 |  |  |  |
| 555 | AC021218.2 |  |  |  |
| 556 | JAG1 |  |  |  |
| 557 | ABCC1 |  |  |  |
| 558 | PHF1 |  |  |  |
| 559 | RAB23 |  |  |  |
| 560 | BICD2 |  |  |  |
| 561 | FKTN |  |  |  |
| 562 | KCNJ6 |  |  |  |
| 563 | RNF150 |  |  |  |
| 564 | AHSA2 |  |  |  |
| 565 | MSX2 |  |  |  |
| 566 | TIAM2 |  |  |  |
| 567 | DHX38 |  |  |  |
| 568 | STEAP3 |  |  |  |
| 569 | KCND2 |  |  |  |
| 570 | COX7A2L |  |  |  |
| 571 | NEUROD1 |  |  |  |
| 572 | KDM3A |  |  |  |
| 573 | ACOT12 |  |  |  |
| 574 | ACSL6 |  |  |  |
| 575 | FAM71C |  |  |  |
| 576 | TCF4 |  |  |  |
| 577 | CCDC91 |  |  |  |
| 578 | SLC7A2 |  |  |  |
| 579 | MN1 |  |  |  |
| 580 | CHRAC1 |  |  |  |
| 581 | KLHL10 |  |  |  |
| 582 | USP37 |  |  |  |
| 583 | TUBB1 |  |  |  |
| 584 | PAX9 |  |  |  |
| 585 | PTAR1 |  |  |  |
| 586 | ELN |  |  |  |
| 587 | FASTKD2 |  |  |  |
| 588 | HEMK1 |  |  |  |
| 589 | RAB37 |  |  |  |
| 590 | SHROOM1 |  |  |  |
| 591 | ACTR3 |  |  |  |
| 592 | CEP57L1 |  |  |  |
| 593 | GOLIM4 |  |  |  |
| 594 | RP5-1052I5.2 |  |  |  |
| 595 | STRAP |  |  |  |
| 596 | SEC14L5 |  |  |  |
| 597 | HPSE |  |  |  |
| 598 | ATMIN |  |  |  |
| 599 | SLC1A2 |  |  |  |
| 600 | CHORDC1 |  |  |  |
| 601 | TOMM20 |  |  |  |
| 602 | ACOT11 |  |  |  |
| 603 | CCDC127 |  |  |  |
| 604 | VGLL3 |  |  |  |
| 605 | ZNF273 |  |  |  |
| 606 | EIF3E |  |  |  |
| 607 | RIN2 |  |  |  |
| 608 | T |  |  |  |
| 609 | FAM171B |  |  |  |
| 610 | ZNF559-ZNF177 |  |  |  |
| 611 | SFPQ |  |  |  |
| 612 | ITM2A |  |  |  |
| 613 | INTS2 |  |  |  |
| 614 | ZNF177 |  |  |  |
| 615 | ZNF692 |  |  |  |
| 616 | PINK1 |  |  |  |
| 617 | EIF4E |  |  |  |
| 618 | FAM219A |  |  |  |
| 619 | ZBTB21 |  |  |  |
| 620 | PSME4 |  |  |  |
| 621 | ARL4A |  |  |  |
| 622 | GPC5 |  |  |  |
| 623 | MFSD4 |  |  |  |
| 624 | NIPAL3 |  |  |  |
| 625 | TLK1 |  |  |  |
| 626 | PCDH17 |  |  |  |
| 627 | SETD4 |  |  |  |
| 628 | PLGLB1 |  |  |  |
| 629 | HMGA1 |  |  |  |
| 630 | C14orf177 |  |  |  |
| 631 | TMPRSS7 |  |  |  |
| 632 | CDK6 |  |  |  |
| 633 | CDC14A |  |  |  |
| 634 | ABCC4 |  |  |  |
| 635 | KRT35 |  |  |  |
| 636 | STT3A |  |  |  |
| 637 | MPZL1 |  |  |  |
| 638 | TRERF1 |  |  |  |
| 639 | TRMT1L |  |  |  |
| 640 | BEND7 |  |  |  |
| 641 | OGFRL1 |  |  |  |
| 642 | CCDC144NL |  |  |  |
| 643 | HSPH1 |  |  |  |
| 644 | DST |  |  |  |
| 645 | LILRB1 |  |  |  |
| 646 | RAP1B |  |  |  |
| 647 | TTC30A |  |  |  |
| 648 | C10orf107 |  |  |  |
| 649 | WASF3 |  |  |  |
| 650 | TFDP2 |  |  |  |
| 651 | DLEU1 |  |  |  |
| 652 | USP38 |  |  |  |
| 653 | CHPF |  |  |  |
| 654 | CSNK1G1 |  |  |  |
| 655 | CHSY1 |  |  |  |
| 656 | ESRRG |  |  |  |
| 657 | RTN1 |  |  |  |
| 658 | TSPYL4 |  |  |  |
| 659 | KCTD12 |  |  |  |
| 660 | SLC6A4 |  |  |  |
| 661 | CLCN6 |  |  |  |
| 662 | TEX15 |  |  |  |
| 663 | SATB1 |  |  |  |
| 664 | NUFIP2 |  |  |  |
| 665 | KMT2C |  |  |  |
| 666 | RP5-1021I20.4 |  |  |  |
| 667 | SYAP1 |  |  |  |
| 668 | GBE1 |  |  |  |
| 669 | ANO5 |  |  |  |
| 670 | TLK2 |  |  |  |
| 671 | HTRA1 |  |  |  |
| 672 | DR1 |  |  |  |
| 673 | RAP1GDS1 |  |  |  |
| 674 | NCAM1 |  |  |  |
| 675 | ISLR2 |  |  |  |
| 676 | C18orf63 |  |  |  |
| 677 | NSMAF |  |  |  |
| 678 | SRSF11 |  |  |  |
| 679 | KLF8 |  |  |  |
| 680 | PRKAA1 |  |  |  |
| 681 | FBXW11 |  |  |  |
| 682 | ZNF528 |  |  |  |
| 683 | CUL4B |  |  |  |
| 684 | ENTPD1 |  |  |  |
| 685 | GPRIN3 |  |  |  |
| 686 | DCAF12L1 |  |  |  |
| 687 | PSD3 |  |  |  |
| 688 | OSGIN2 |  |  |  |
| 689 | RBM12B |  |  |  |
| 690 | SMG9 |  |  |  |
| 691 | TMEM257 |  |  |  |
| 692 | IKBIP |  |  |  |
| 693 | KLHL29 |  |  |  |
| 694 | JMJD1C |  |  |  |
| 695 | KIAA0232 |  |  |  |
| 696 | ZC3H12B |  |  |  |
| 697 | XPOT |  |  |  |
| 698 | FNDC3B |  |  |  |
| 699 | CREBRF |  |  |  |
| 700 | PLGLB2 |  |  |  |
| 701 | COX6B2 |  |  |  |
| 702 | FAM203A |  |  |  |
| 703 | ZNF470 |  |  |  |
| 704 | SLC26A7 |  |  |  |
| 705 | CHRNB1 |  |  |  |
| 706 | LRRIQ3 |  |  |  |
| 707 | SH3BP4 |  |  |  |
| 708 | FBXO43 |  |  |  |
| 709 | MMGT1 |  |  |  |
| 710 | SLC26A11 |  |  |  |
| 711 | UBR1 |  |  |  |
| 712 | LRRC58 |  |  |  |
| 713 | GLRB |  |  |  |
| 714 | RXFP1 |  |  |  |
| 715 | INO80D |  |  |  |
| 716 | C12orf23 |  |  |  |
| 717 | CEP68 |  |  |  |
| 718 | BACH1 |  |  |  |
| 719 | PTPN4 |  |  |  |
| 720 | SCN2A |  |  |  |
| 721 | HAS2 |  |  |  |
| 722 | ZNF286A |  |  |  |
| 723 | MAP4K3 |  |  |  |
| 724 | RSL24D1 |  |  |  |
| 725 | EIF2AK2 |  |  |  |
| 726 | FAM200A |  |  |  |
| 727 | PDLIM3 |  |  |  |
| 728 | BTG1 |  |  |  |
| 729 | ZDHHC17 |  |  |  |
| 730 | BAIAP2 |  |  |  |
| 731 | TBC1D30 |  |  |  |
| 732 | CTD-2368P22.1 |  |  |  |
| 733 | YBX3 |  |  |  |
| 734 | ERCC6L2 |  |  |  |
| 735 | BDKRB2 |  |  |  |
| 736 | PCNA |  |  |  |
| 737 | C20orf202 |  |  |  |
| 738 | DNAH14 |  |  |  |
| 739 | NOM1 |  |  |  |
| 740 | KANSL2 |  |  |  |
| 741 | PTRF |  |  |  |
| 742 | IKZF5 |  |  |  |
| 743 | SMARCC1 |  |  |  |
| 744 | MAMDC2 |  |  |  |
| 745 | SORT1 |  |  |  |
| 746 | BCLAF1 |  |  |  |
| 747 | SETD2 |  |  |  |
| 748 | DSEL |  |  |  |
| 749 | PSTPIP2 |  |  |  |
| 750 | CLEC7A |  |  |  |
| 751 | MTUS1 |  |  |  |
| 752 | CDYL |  |  |  |
| 753 | THRA |  |  |  |
| 754 | ELMOD1 |  |  |  |
| 755 | KLHL5 |  |  |  |
| 756 | TARDBP |  |  |  |
| 757 | OTUD3 |  |  |  |
| 758 | APAF1 |  |  |  |
| 759 | CREB5 |  |  |  |
| 760 | UBE2K |  |  |  |
| 761 | ITGB1BP1 |  |  |  |
| 762 | ADSS |  |  |  |
| 763 | AL953854.2 |  |  |  |
| 764 | PTCH1 |  |  |  |
| 765 | QSER1 |  |  |  |
| 766 | KIN |  |  |  |
| 767 | NFIC |  |  |  |
| 768 | FNDC1 |  |  |  |
| 769 | SPDL1 |  |  |  |
| 770 | TARBP2 |  |  |  |
| 771 | RPGRIP1L |  |  |  |
| 772 | RAC1 |  |  |  |
| 773 | ACER2 |  |  |  |
| 774 | PSMF1 |  |  |  |
| 775 | SNX1 |  |  |  |
| 776 | MBNL3 |  |  |  |
| 777 | UTRN |  |  |  |
| 778 | TBP |  |  |  |
| 779 | TECTB |  |  |  |
| 780 | TMX4 |  |  |  |
| 781 | TWF1 |  |  |  |
| 782 | YAF2 |  |  |  |
| 783 | ELOVL4 |  |  |  |
| 784 | LMAN1 |  |  |  |
| 785 | SLC5A12 |  |  |  |
| 786 | KCTD20 |  |  |  |
| 787 | GEMIN5 |  |  |  |
| 788 | TMEM135 |  |  |  |
| 789 | RP11-690P14.4 |  |  |  |
| 790 | HBS1L |  |  |  |
| 791 | GRB10 |  |  |  |
| 792 | SASS6 |  |  |  |
| 793 | BLOC1S6 |  |  |  |
| 794 | VPS29 |  |  |  |
| 795 | TMEM50B |  |  |  |
| 796 | USP8 |  |  |  |
| 797 | ZKSCAN5 |  |  |  |
| 798 | CSMD3 |  |  |  |
| 799 | DPP4 |  |  |  |
| 800 | CSMD2 |  |  |  |
| 801 | RALGPS1 |  |  |  |
| 802 | PSMA2 |  |  |  |
| 803 | ZEB1 |  |  |  |
| 804 | SLC1A1 |  |  |  |
| 805 | ZNF432 |  |  |  |
| 806 | MYEF2 |  |  |  |
| 807 | GMIP |  |  |  |
| 808 | TMEM47 |  |  |  |
| 809 | CDH10 |  |  |  |
| 810 | PTCD3 |  |  |  |
| 811 | ATP6V1A |  |  |  |
| 812 | SLC38A2 |  |  |  |
| 813 | HELZ |  |  |  |
| 814 | WNT5A |  |  |  |
| 815 | C14orf39 |  |  |  |
| 816 | RAB3IP |  |  |  |
| 817 | LRRC23 |  |  |  |
| 818 | HNRNPA1 |  |  |  |
| 819 | FAM60A |  |  |  |
| 820 | SETD8 |  |  |  |
| 821 | TRIM13 |  |  |  |
| 822 | FAM203B |  |  |  |
| 823 | ZMYND11 |  |  |  |
| 824 | ESR2 |  |  |  |
| 825 | PLK2 |  |  |  |
| 826 | DLX3 |  |  |  |
| 827 | ZNF253 |  |  |  |
| 828 | ADAMTS5 |  |  |  |
| 829 | MORC3 |  |  |  |
| 830 | ACSL4 |  |  |  |
| 831 | HOMER2 |  |  |  |
| 832 | NRK |  |  |  |
| 833 | GRHL1 |  |  |  |
| 834 | SYF2 |  |  |  |
| 835 | COL4A5 |  |  |  |
| 836 | GDAP1 |  |  |  |
| 837 | KCNT1 |  |  |  |
| 838 | C10orf32 |  |  |  |
| 839 | C11orf30 |  |  |  |
| 840 | C2orf73 |  |  |  |
| 841 | THSD7A |  |  |  |
| 842 | FGD4 |  |  |  |
| 843 | FARSB |  |  |  |
| 844 | GNAQ |  |  |  |
| 845 | RAB27A |  |  |  |
| 846 | TXNL1 |  |  |  |
| 847 | CCDC38 |  |  |  |
| 848 | CTD-2132N18.3 |  |  |  |
| 849 | SLC5A1 |  |  |  |
| 850 | GCNT1 |  |  |  |
| 851 | HCCS |  |  |  |
| 852 | NDUFB2 |  |  |  |
| 853 | HIVEP3 |  |  |  |
| 854 | KIAA0408 |  |  |  |
| 855 | F2R |  |  |  |
| 856 | GJC1 |  |  |  |
| 857 | ZBTB5 |  |  |  |
| 858 | ZNF100 |  |  |  |
| 859 | BCL2 |  |  |  |
| 860 | GOLGA8F |  |  |  |
| 861 | MED7 |  |  |  |
| 862 | EP300 |  |  |  |
| 863 | PPP2R5A |  |  |  |
| 864 | CRBN |  |  |  |
| 865 | RICTOR |  |  |  |
| 866 | EPS15 |  |  |  |
| 867 | LAMTOR3 |  |  |  |
| 868 | MURC |  |  |  |
| 869 | MSH6 |  |  |  |
| 870 | CLHC1 |  |  |  |
| 871 | HLTF |  |  |  |
| 872 | DNMT1 |  |  |  |
| 873 | ZNF594 |  |  |  |
| 874 | ITSN1 |  |  |  |
| 875 | CTNNB1 |  |  |  |
| 876 | CNTNAP3B |  |  |  |
| 877 | MAP4K4 |  |  |  |
| 878 | SOAT1 |  |  |  |
| 879 | CAPS2 |  |  |  |
| 880 | ARL11 |  |  |  |
| 881 | SMU1 |  |  |  |
| 882 | ARHGAP19 |  |  |  |
| 883 | TRPS1 |  |  |  |
| 884 | IP6K3 |  |  |  |
| 885 | LMBR1 |  |  |  |
| 886 | ATP2C1 |  |  |  |
| 887 | NCOA7 |  |  |  |
| 888 | NFIA |  |  |  |
| 889 | PDPN |  |  |  |
| 890 | GNPTAB |  |  |  |
| 891 | FXR1 |  |  |  |
| 892 | ONECUT2 |  |  |  |
| 893 | TUBB3 |  |  |  |
| 894 | RSRC2 |  |  |  |
| 895 | LRRC8B |  |  |  |
| 896 | ABCC9 |  |  |  |
| 897 | SMIM14 |  |  |  |
| 898 | MCTP2 |  |  |  |
| 899 | COL6A6 |  |  |  |
| 900 | CDON |  |  |  |
| 901 | IPO11 |  |  |  |
| 902 | PDHX |  |  |  |
| 903 | FGF2 |  |  |  |
| 904 | MBOAT2 |  |  |  |
| 905 | CTC-432M15.3 |  |  |  |
| 906 | CSF1R |  |  |  |
| 907 | CAMK2D |  |  |  |
| 908 | FAM126A |  |  |  |
| 909 | SLC19A2 |  |  |  |
| 910 | BEND6 |  |  |  |
| 911 | KLHL24 |  |  |  |
| 912 | ZNF684 |  |  |  |
| 913 | ELMO2 |  |  |  |
| 914 | SHE |  |  |  |
| 915 | UMPS |  |  |  |
| 916 | HAPLN1 |  |  |  |
| 917 | LRPAP1 |  |  |  |
| 918 | ZC3H14 |  |  |  |
| 919 | DTL |  |  |  |
| 920 | C15orf40 |  |  |  |
| 921 | TEX101 |  |  |  |
| 922 | ATG5 |  |  |  |
| 923 | ROCK1 |  |  |  |
| 924 | KIAA1958 |  |  |  |
| 925 | CHD9 |  |  |  |
| 926 | INO80C |  |  |  |
| 927 | MRE11A |  |  |  |
| 928 | ZNF45 |  |  |  |
| 929 | SUZ12 |  |  |  |
| 930 | FNTA |  |  |  |
| 931 | YES1 |  |  |  |
| 932 | MED13 |  |  |  |
| 933 | MEMO1 |  |  |  |
| 934 | ERLIN2 |  |  |  |
| 935 | YIPF6 |  |  |  |
| 936 | STRBP |  |  |  |
| 937 | VWA5A |  |  |  |
| 938 | ARL4C |  |  |  |
| 939 | CDH1 |  |  |  |
